# Determinants of survival after re-resection for recurrent glioblastoma: a meta-analysis

**DOI:** 10.1007/s00701-025-06755-6

**Published:** 2026-01-13

**Authors:** Manuel V. Baby, Rithvik M. Narendranath, Symriti Kaur-Paneser, Daniele S. C. Ramsay, Hariharan Subbiah Ponniah, Srikar R. Namireddy, Ahmed Salih, Ahkash Thavarajasingam, Daniel Scurtu, Andreas Kramer, Veit Stöcklein, Darius Kalasauskas, Dragan Jankovic, Florian Ringel, Santhosh G. Thavarajasingam

**Affiliations:** 1 Imperial Brain and Spine Initiative, Imperial College London, London, UK; 2 Faculty of Medicine, Imperial College London, London, UK; 3 Faculty of Medicine, Medizinische Hochschule Hannover, Hannover, Germany; 4 Department of Neurosurgery, University Medical Center Mainz, Mainz, Germany; 5 Department of Neurosurgery, LMU University Hospital, LMU Munich, Munich, Germany

**Keywords:** Glioblastoma, Prognostic, Re-resection, Recurrence, Survival

## Abstract

**Purpose:**

Glioblastoma (GBM) inevitably recurs despite maximal safe resection and standard chemoradiotherapy. The factors influencing survival after first recurrence and re-resection remain controversial.

**Research question:**

What are the prognostic factors influencing survival following re-resection of glioblastoma?

**Methods:**

A systematic search of major databases was conducted for original studies reporting on survival outcomes. Data on hazard ratios (HR) for overall survival and key prognostic factors were extracted, followed by meta-analyses of univariate and multivariate Cox models. Study quality and risk of bias were assessed.

**Results:**

A total of 30 studies were included. Gross total resection and methylated MGMT promoter status were significantly associated with improved survival, with pooled HRs of 0.52 (95% CI: 0.36–0.76, *p* < 0.001) and 0.58 (95% CI: 0.45–0.75, *p* < 0.001), respectively. In contrast, age was modestly associated with worse survival (HR: 1.02, 95% CI: 1.01–1.03, *p* < 0.001). Preoperative Karnofsky Performance Status (KPS) < 70 was associated with worse survival (HR: 2.25, 95% CI: 1.59–3.19, *p* < 0.001). Adjuvant chemotherapy (HR: 0.69, 95% CI: 0.33–1.45, *p* = 0.33) and time to re-resection (HR: 0.69, 95% CI: 0.41–1.16, *p* = 0.16) failed to show consistent survival benefits.

**Conclusion:**

Our findings suggest gross total resection of contrast-enhancing tumour and MGMT promoter methylation are strongly associated with improved survival following first recurrence of glioblastoma. Conversely, age, preoperative KPS, adjuvant chemotherapy, and timing of re-resection showed inconsistent or non-significant associations, emphasizing the need for prospective studies to refine prognostic assessments and guide individualized treatment strategies in recurrent glioblastoma.

**Supplementary information:**

The online version contains supplementary material available at 10.1007/s00701-025-06755-6.

## Highlights

- Re-resection should be considered where gross total re-resection is feasible.
- Methylated MGMT promoter status indicates effectiveness of alkylating agents in recurrent glioblastoma.
- More congruence in study design and outcome reporting on KPS and time to re-resection is required to conclude on their prognostic influence.

**Supplementary information:**

The online version contains supplementary material available at 10.1007/s00701-025-06755-6.

## Introduction

Glioblastoma (GBM), or grade 4 glioma as per the WHO classification, is the most common primary malignant brain tumour, with an annual incidence of approximately 3.2 per 100,000 people [16]. The current standard of care at initial diagnosis involves maximal safe resection followed by radiotherapy and concomitant and adjuvant temozolomide, known as the Stupp protocol [23]. This regimen has been shown to extend median survival by about 2.5 months compared to radiotherapy alone [23]. The extent of resection during initial surgery is a well-recognized prognostic factor, with gross total resection providing the most substantial survival benefit [5].

Despite optimal multimodal therapy, GBM almost invariably recurs, with a median progression-free survival of approximately 6.9 months [42].

The management of recurrent GBM is challenging and lacks a standardized approach. The Response Assessment in Neuro-Oncology (RANO) criteria have refined the definition of tumour progression beyond the traditional Macdonald criteria, incorporating the presence of new lesions, increased T2/FLAIR signal intensity, clinical deterioration attributable to the tumour, and/or increased corticosteroid requirements [22, 31]. Repeat resection is considered in 10% to 30% of patients meeting these progression criteria [45]. However, unlike the standardized initial treatment, the role of re-resection at recurrence remains controversial. The European Association of Neuro-Oncology (EANO) guidelines suggest a range of treatment options for recurrent GBM, including nitrosoureas, additional temozolomide, bevacizumab, and repeat radiation, tailored according to patient factors such as Karnofsky Performance Status (KPS), neurological function, age, and previous treatment history [19]. Nevertheless, there is no clear consensus on the optimal management strategy for recurrence, and the survival benefit of re-resection remains uncertain.

Previous meta-analyses have reported a potential association between repeat resection and improved survival in recurrent GBM [56]. However, these analyses did not provide a detailed quantitative assessment of individual prognostic factors. Our meta-analysis provides a comprehensive, quantitative assessment of key variables, and seeks to identify which patients are most likely to benefit from re-resection, ultimately supporting more personalized treatment strategies for recurrent GBM.

## Methods

### Search strategy and selection criteria

This systematic review was conducted following guidelines outlined by the Cochrane Collaboration and registered on PROSPERO (CRD 42024500376). The Preferred Reporting Items for Systematic Reviews and Meta-Analyses (PRISMA) 2020 statement can be found in Supplementary Digital Content: Table 1. A comprehensive search of the literature was performed on January 14, 2024, across four major databases: Medline, EMBASE, PubMed, and Scopus. The search strategy aimed to identify original studies investigating a range of prognostic factors associated with survival following re-resection for recurrent glioblastoma. The full search strategy can be found in Supplementary Digital Content: Table 2. The Covidence tool was utilized to manage study selection and resolve conflicts [1]. Two independent reviewers (SKP and RMV) screened the titles and abstracts for eligible studies. Disagreements were resolved by a third reviewer (MVB). Studies were included if they reported on at least a subset of patients undergoing re-resection for glioblastoma progression and compared two or more groups based on predefined prognostic factors. Re-resection was defined as a second surgical intervention aimed at removing or debulking a recurrent glioblastoma following initial surgery. Studies that conflated outcome data with lower-grade gliomas (e.g., anaplastic astrocytoma or low-grade gliomas) were excluded to ensure consistency in the patient cohort. Full inclusion and exclusion criteria can be found in Supplementary Digital Content: Table 3.

### Objectives

This review sought to answer the following key research question:

- What are the prognostic factors influencing survival following re-resection of glioblastoma?

### Data extraction and quality assessment

Data extraction was performed manually using a standardized Excel spreadsheet, with all extracted data cross-verified against the original articles. Risk of bias was assessed using the ROBINS-I tool across all seven domains, with two reviewers (SKP and RMV) independently appraising each study and resolving discrepancies through discussion with a third reviewer (MVB) [44]. A list of all extracted variables can be found in Supplementary Digital Content: Table 4.

### Data analysis

Statistical analysis and forest plot synthesis were conducted using the meta and metafor packages in R (version 4.4.1) [52]. Meta-analyses were performed on studies reporting Cox proportional hazards ratios (HRs) for survival across the investigated prognostic factors. Both univariate and multivariate pooled HR estimates were computed when data were available, using a random effects model to account for significant heterogeneity. The Cox regression model was selected as it allows for the evaluation of both quantitative factors (e.g., age) and categorical variables (e.g., extent of resection, MGMT promoter methylation status). An HR < 1.00 indicates an association with increased survival, while an HR > 1.00 indicates worse survival [18, 43]. Heterogeneity was assessed using the I^2^ statistic, and standard errors were calculated based on the 95% confidence intervals provided alongside the Cox HRs, following the formula by Parmar et al. [32]. The level of evidence was scored using the ROBINS-I tool and Oxford Centre of Evidence-Based Medicine (OCEBM) Levels of Evidence. Results of the bias assessment and evidence levels can be found in Supplementary Digital Content: Table 5 and 6, respectively. All statistical analyses were performed using R (version 4.4.1), and the detailed analysis code is available in Supplementary Digital Content: Table 7.

### Sensitivity analysis for IDH-wildtype patients

The 2021 WHO classification defines glioblastoma as an Isocitrate dehydrogenase (IDH) wildtype grade 4 glioma [12]. Many studies on recurrent glioblastoma predate this revision and often did not report IDH status, including both IDH-wildtype and IDH-mutant cases, though the majority were IDH-wildtype. To address this, we conducted a sensitivity analysis by repeating the meta-analysis and forest plot synthesis, where feasible, restricted to studies that included exclusively IDH-wildtype patients for the identified prognostic factors.

In accordance with the WHO 2021 diagnostic framework, molecular glioblastomas (IDH-wildtype tumours meeting molecular GBM criteria even in the absence of histological features such as necrosis or microvascular proliferation) were included within the glioblastoma cohort whenever explicitly identified in the source studies. However, as most of the included studies predated the molecular classification, they did not distinguish between molecular and histologically defined GBM.

## Results

**Fig. 1:**
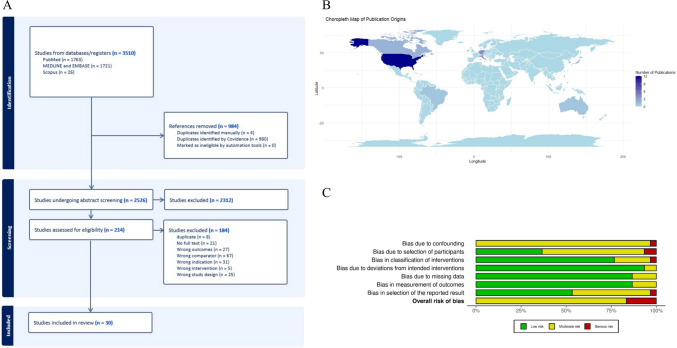
**A** The PRISMA flowchart outlining the study selection process. Studies were excluded if the endpoints measured were non-survival outcomes (wrong outcomes), if patients receiving re-resection at recurrence were compared with those not receiving re-resection (wrong comparators), if they included patients with lower-grade gliomas in their cohort (wrong indication), if treatments such as stereotactic radiorsurgery or medication such as bevacizumab at recurrence were assessed alone (wrong intervention), or if they were case reports or series (wrong study design). **B** A world map showing the origin of published studies. Darker shades of blue indicate a higher proportion of studies originating from the country. Countries represented include Australia (*n* = 1), Brazil (*n* = 1), Canada (*n* = 2), Czechia (*n* = 1), Germany (n = 4), Hong Kong (*n* = 1), Italy (*n* = 4), Japan (*n* = 2), the Netherlands (*n* = 1), South Korea (*n* = 1), Switzerland (*n* = 1) and the USA (*n* = 12). **C** A risk of bias summary plot displaying the distribution of risk-of-bias judgements for all included studies (*n* = 30) [3, 4, 6, 7, 9–11, 13–15, 17, 20, 21, 25, 27, 29, 30, 33, 34, 36–38, 46–48, 50, 51, 53, 54, 57] as determined using the ROBINS-I tool. The summary plot and a traffic light plot shown in supplementary Fig. 1 was generated using the web-app *robvis* [40]

A total of 3,510 studies were screened, with 214 full-text articles assessed against the inclusion and exclusion criteria. Ultimately, 30 studies met the eligibility criteria for inclusion in this systematic review, of which 18 studies were included in the meta-analysis (Fig. 1A). The combined sample size for the systematic review was 3,314 patients, and the pooled sample size for the meta-analysis comprised 1,741 patients. Detailed characteristics of the included studies are summarized in Table 1, and a geographic distribution of study origins is presented in Fig. 1B. Out of the 30 included studies, 25 were assessed as having a ‘moderate’ risk of bias, while five had a ‘serious’ risk of bias, according to the ROBINS-I tool (Supplementary Digital Content: Table 6, Fig. 1). A summary of the risk of bias across all seven domains is provided in Fig. 1C. Based on OCEBM guidance, all 30 studies were classified as level 3b evidence (Supplementary Digital Content: Table 5). A summary of the key findings of the included studies is shown in Table 2.

**Table 1:** Study characteristics of the included studies in this systematic review

Study	Sample Size (*n *=)	Study type	Country	Percentage of known IDH-wildtype patients (%)	Adjuvant therapy following Initial Resection (IR)	Tumour Recurrence (*n* =)	Treatment Related Changes (TRC)/Pseudoprogression (*n* =)	Adjuvant therapy following 1RR
Bagley et al. 2019	37	Retrospective study	USA	100	RTx & TMZ	All patients	None defined	Not mentioned
Barz et al. 2022	123	Retrospective study	Germany	100	Not mentioned	All patients	None defined	RTx & TMZ CTx alone RTx alone
Bloch et al. 2012	107	Retrospective study	USA	Not reported	RTx & TMZ	All patients	None defined	CTx (Irinotecan, lomustine)
Brandes et al. 2016	270	Retrospective study	Italy	Not reported	RTx & TMZ	All patients	None defined	CTx (TMZ and nitrosoureas)
Dalle Ore et al. 2019	110	Retrospective study	USA	57.3 (IDH status only known for 70/110 patients)	RTx & TMZ	All patients	None defined	CTx (TMZ)
De Bonis et al. 2013	76^*^	Retrospective study	Italy	Not reported	RTx & TMZ	All patients	None defined	CTx (TMZ, cisplatin, fotemustine, carmustine, irinotecan) RTx
Goldman et al. 2018	163	Retrospective study	USA	Not reported	Neoadjuvant CTx RTx & TMZ Carmustine	All patients	None defined	CTx (TMZ, carmustine)
Hennessy et al. 2022	32	Retrospective Study	Ireland	Not reported	RTx & TMZ	All patients	None defined	CTx and RTx
Kalita et al. 2023	106	Retrospective study	Czechia	88.2	RTx & TMZ CTx alone RTx alone	All patients	None (part of exclusion criteria)	CTx alone RTx alone CTx and RTx
Mandl et al. 2008	20	Retrospective study	The Netherlands	Not reported	Not mentioned	All patients	None defined	CTx alone SRT
McNamara et al. 2014	107	Retrospective study	Canada; Australia	Not reported	RTx & TMZ RTx alone TMZ alone Dexamethasone	All patients	None defined	CTx (TMZ, lomustine, oral etoposide, others)
Melnick et al. 2022	115	Retrospective study	USA	94.1	Not mentioned	106	9	RTx & TMZ RTx alone TMZ alone
Montemurro et al. 2021	63	Retrospective study	Italy	98.1	Not mentioned	All patients	None defined	CTx
Okita et al. 2012	32	Retrospective study	Japan	Not reported	RTx CTx (TMZ, ACNU nimustine hydrochloride)	All patients	None defined	Not mentioned
Oppenlander et al. 2014	170	Retrospective study	USA	Not reported	RTx & CTx	All patients	None defined	CTx and RTx
Park et al. 2013	55	Retrospective study	South Korea	Not reported	RTx & TMZ or nimustine	All patients	None defined	CTx
Patrizz et al. 2021	137	Retrospective study	USA	Not reported	TMZ Bevacizumab Gamma knife surgery	115	22	RTx CTx (TMZ, irinotecan, BCNU, lomustine)
Perrini et al. 2017	48	Retrospective study	Italy	Not reported	Not mentioned	All patients	None defined	RTx & TMZ CTx (fotemustine, TMZ)
Pessina et al. 2017	64	Retrospective study	USA	100	Not mentioned	All patients	None (part of exclusion criteria)	RTx & CTx CTx alone RTx alone
Pinsker et al. 2001	38	Retrospective study	Germany	Not reported	Not mentioned	All patients	None defined	RTx
Quick et al. 2014	40	Retrospective study	USA	Not reported	RTx & TMZ	All patients	None (part of exclusion criteria)	RTx & TMZ RTx CTx (TMZ, CCNU, ACNU)
Ringel et al. 2016	503	Retrospective study	Germany	Not reported	RTx alone CTx alone RTx & CTx	All patients	None defined	RTx & CTx RTx alone CTx (TMZ, ACNU, BCNU, CCNU) Other experimental therapies
Sonoda et al. 2014	61	Retrospective study	Japan	Not reported	RTx & TMZ/nitrosourea	All patients	None defined	SRT CTx (TMZ, ifosfamide + cisplatin + etoposide/intrathecal methotrexate)
Suchorska et al. 2016	71	Prospective cohort study	Germany; Switzerland	Not reported	Not mentioned	All patients	None defined	Not mentioned
Voisin et al. 2022	87	Retrospective study	Canada	100	RTx & TMZ TMZ alone	All patients	None defined	RTx & TMZ TMZ alone
Woo et al. 2023	137	Retrospective study	Hong Kong	90.0	RTX & TMZ	All patients	None defined	RTx CTx (TMZ, CCNU, PCV)
Woodroffe et al. 2020	37	Retrospective study	USA	96.9	RTx & CTx	All patients	None (part of exclusion criteria)	RTX & CTx (TMZ)
Woodworth et al. 2013	59	Retrospective study	USA	Not reported	Not mentioned	42	17	RTx & CTx CTx alone (Gliadel, temodar) RTx alone
Yong et al. 2014	97	Retrospective study	USA	Not reported	Not mentioned	All patients	None defined	Rtx and Ctx
Zanovello et al. 2016	39	Retrospective study	Brazil	Not reported	Not mentioned	All patients	None defined	RTx alone CTx (BCNU, TMZ, PCV)

Table 1 outlines the characteristics of the included studies in this systematic review (*n *= 30). Key variables including the study author and date of publication, sample size along with gender composition, study type/design, country, range of treatments offered alongside initial resection, number of patients undergoing repeat resection for treatment related changes (TRC) or pseudo-progression vs for recurrent glioblastoma, and range of adjuvant treatments offered alongside repeat resection have been tabulated for each study. The abbreviations used in the table are as follows: first re-resection (1RR), radiotherapy (*RTx)*, temozolomide (*TMZ)*, chemotherapy (*CTx)*, stereotactic radiotherapy (*SRT)*, nimustine hydrochloride alkylating agents (*ACNU/BCNU/CCNU)*, treatment regimen for recurrent glioblastoma comprising procarbazine, lomustine and vincristine (*PCV)*

**Table 2:** Summary of the prognostic factors studied, reported survival outcomes and main conclusions of the included studies

Study	Period of follow-up	Median time to recurrence/re-resection from point of initial resection (months)	Prognostic factors studied	Survival measures included	Main Conclusions
Bagley et al. 2019	2013–2016	To re-resection: 8.8	Age at 1RR TTR Sex WHO performance status Extent of Resection MGMT methylation status	Overall survival Cox hazard ratios	Increased Ki67 proliferation index, shorter time period between IR and 1RR and WHO PS 2–4 were associated with worse overall survival in repeat resection of GBM
Barz et al. 2022	2007–2010	Not mentioned	Age at 1RR Preoperative KPS Extent of Resection	Survival after re-resection Cox hazard ratios	Preoperative KPS (>/< = 80) and EOR significantly associated with survival
Bloch et al. 2012	2005—2009	Not mentioned	Age at 1RR Extent of Resection (at initial and repeat resection) Preoperative KPS Eloquent tumour location Adjuvant chemotherapy	Overall Survival Survival after re-resection Cox hazard ratios	GTR at re-resection can compensate for incomplete initial resection, using intraoperative adjuncts and imaging
Brandes et al. 2016	2005–2014	Not mentioned	Age at 1RR Extent of Resection Adjuvant chemotherapy MGMT methylation status	Survival after re-resection Cox hazard ratios	GTR, MGMT methylation and younger age associated with improved survival following 1RR
Dalle Ore et al. 2019	2008–2015	To re-resection: 12.9	Extent of Resection Adjuvant therapy Time to re-resection	Cox hazard ratios	Treatment with bevacizumab, time to reoperation, presence of sarcoma at reoperation significantly associated with survival
De Bonis et al. 2013	2002—2008	Not mentioned	Extent of re-resection Adjuvant therapy Preoperative KPS	Overall Survival Cox hazard ratios	KPS < 70 significant (including patients not receiving 1RR at recurrence). Adjuvant therapy at 1RR also significant. EOR, younger age not significant
Goldman et al. 2018	2005–2014	Not mentioned	Age at 1RR Time to re-resection Sex Multifocality of tumour Critical/eloquent tumour areas Preoperative KPS Extent of Resection Adjuvant therapy	Cox hazard ratios	Time to re-resection, age, sex, EOR at both IR and 1RR, KPS, multifocality, eloquent region of tumour are identified prognostic factors. Accounting for time to re-resection reduces positive association between repeat surgery and survival
Hennessy et al. 2022	2015—2018	To re-resection: 13.5	Age at 1RR Sex MGMT methylation status Preoperative KPS Tumour location Time to re-resection Extent of Resection	Overall Survival Survival after re-resection	MGMT methylated status and a longer interval between initial and repeat resections confers significantly improved overall survival
Kalita et al. 2023	2008—2019	To recurrence: 10.1	Time to progression/recurrence Sex IDH mutation status Adjuvant therapy MGMT methylation status	Survival after re-resection Cox hazard ratios	Repeat re-resection, if done within a minimum time since initial diagnosis/surgery, can have a positive effect on survival
Mandl et al. 2008	1999–2005	Not mentioned	Adjuvant therapy	Survival after re-resection	Patients with symptomatic recurrent GBM with severe mass effect should only receive 1RR if followed by adjuvant therapy
McNamara et al. 2014	2004—2011	To re-resection: 11.5	Time to progression/recurrence Time to re-resection	Overall Survival Survival after re-resection Time ratio	Time to progression associated with survival, alongside NLR > 4.0 and adjuvant systemic therapy
Melnick et al. 2022	2011–2019	To re-resection: 19.6	Age at 1RR Preoperative KPS status IDH mutation status MGMT methylation status	Overall Survival Survival after re-resection	MGMT methylation significant for better OS, histological tumour recurrence significant for worse survival from 1RR (but not overall survival)
Montemurro et al. 2021	2006–2020	To recurrence: 10.0	Age at 1RR Time to progression/recurrence Sex Tumour volume Tumour location Extent of Resection (at initial and repeat resection) Adjuvant chemotherapy MGMT methylation status Molecular profile	Overall Survival Progression-free survival Cox hazard ratios	EOR at first and second recurrence is an important prognostic factor for survival. PFS, female sex, MGMT methylation, and adjuvant therapy at recurrence are also significant prognostic factors
Okita et al. 2012	1996–2010	Not mentioned	MIB-1 index MGMT methylation status	Survival after re-resection Cox hazard ratio	MIB-1 index at second surgery only found to be significant factor in multivariate analysis, MGMT methylation insignificant. MGMT status frequently observed to change
Oppenlander et al. 2014	2001—2011	Not mentioned	Age Preoperative KPS Extent of Resection	Overall Survival Cox hazard ratio	Increasing extent of resection above 80% associated with survival benefit at re-resection, but also risk of transient neurological complications
Park et al. 2013	2000–2010	Not mentioned	Tumour volume Ependymal involvement Preoperative KPS Extent of Resection Adjuvant therapy	Overall Survival Cox hazard ratio	Significant association reported for KPS > 70, ependymal involvement, adjuvant treatment
Patrizz et al. 2021	2005–2020	Not mentioned	Treatment related changes Vs Tumour recurrence	Overall Survival Survival after re-resection Progression-free survival	No significant difference in survival between histologically diagnosed pseudo-progression and tumour recurrence
Perrini et al. 2017	2011–2015	Not mentioned	Age at 1RR Sex Tumour location Preoperative KPS Extent of resection (at initial and repeat Adjuvant chemotherapy	Overall Survival Survival after re-resection Cox hazard ratio	EOR at recurrence significantly predicts survival outcome; GTR at 1RR especially following GTR at IR (not significant on multi analysis). Craniotomy should be offered at recurrence for people with good pre-op KPS
Pessina et al. 2017	2008–2014	To recurrence: 17	Age at 1RR Residual tumour volume Preoperative KPS Extent of resection Adjuvant therapy	Overall Survival	Younger age, preop KPS > = 90, reduced residual tumour volume and adjuvant therapy found to be significant; EOR, MGMT status insignificant. However, selection criterion for repeat surgery was KPS > 70, age > 70
Pinsker et al. 2001	1993—1998	To re-resection: 10.5	Age at 1RR Time to re-resection Sex Tumour location Preoperative KPS Extent of Resection	Overall Survival Survival after re-resection	Overall survival from diagnosis is affected by age < 50 although > 70 pts not reoperated, extent of resection, and a longer period of recurrence-free survival (indicated by time from IR to 1RR). KPS > 80 also prolongs survival
Quick et al. 2014	2007–2010	To re-resection: 10.2	Age at 1RR Time to progression/recurrence Tumour volume Preoperative KPS Extent of Resection MGMT methylation status	Overall Survival Survival after re-resection	GTR associated with better survival following 1RR
Ringel et al. 2016	2006–2015	To re-resection: 9.1	Age at 1RR Time to re-resection Tumour location Preoperative KPS Extent of resection (at initial and repeat resection) Adjuvant therapy	Overall survival	Age, extent of resection at first re-resection and adjuvant therapy significant for improved survival. Complete re-resection significantly associated with improved survival
Sonoda et al. 2014	1997—2010	Not mentioned	Age at 1RR Sex Tumour volume Subventricular zone involvement Preoperative KPS Extent of Resection Adjuvant radiotherapy	Overall Survival Survival after re-resection	Subventricular zone tumour involvement significantly associated with worse survival after 1RR
Suchorska et al. 2016	2015 – (DIRECTOR Trial)	To recurrence: 11.5	Extent of Resection	Survival after re-resection	GTR improves both post-recurrence survival and quality of life in patients following 1RR
Voisin et al. 2022	2011–2021	To recurrence: 12.4	Age at 1RR Time to progression/recurrence Sex Preoperative KPS Extent of Resection (at initial and repeat resection)	Overall Survival Survival after re-resection Cox hazard ratio	Patients with more than six months between IR and recurrence benefit most from re-operation
Woo et al. 2023	2006—2020	Not mentioned	Tumour volume Tumour location Preoperative KPS Extent of Resection 5-ALA fluorescence guided repeat resection Adjuvant therapy	Survival after re-resection	NIH Recurrent GBM scale 'seems to offer' reliable prognostic indication in terms of post-progression survival
Woodroffe et al. 2020	2007—2017	Not mentioned	Sex Tumour volume/FLAIR volume Tumour location Preoperative KPS Extent of Resection	Overall Survival Progression-free survival Cox hazard ratio	Tumour volume (volume of enhancement) and presence of critical/eloquent areas have a significant association with survival
Woodworth et al. 2013	1996–2013	To re-resection: 9.0	Age at 1RR Time to re-resection Sex Tumour volume Tumour location Preoperative KPS Extent of Resection Adjuvant radiotherapy	Cox hazard ratio	Pathological criteria need to be determined to distinguish between active/recurrent GBM and pseudo-progression
Yong et al. 2014	2002—2012	Not mentioned	Age Sex Tumour location Preoperative KPS Extent of resection Tumour regrowth rate	Overall survival Survival after re-resection Cox hazard ratios	Maximal extent of resection should be aimed for with patients undergoing re-resection
Zanovello et al. 2016	2000—2015	To re-resection: 4.7	Age at 1RR Time to re-resection Sex Recurrence at distant location Tumour location Preoperative KPS Extent of Resection (at initial and repeat resection) Adjuvant therapy	Overall Survival Relative Risk of mortality	Adjuvant treatment performance correlates with survival in reoperated GBM, as does EOR both in univariate and multivariate analyses

Table 2 summarises the prognostic factors studied, including for some studies factors linked to the initial resection and the median/mean time interval between initial resection of tumour and either recurrence or re-resection. Also reported is the period during which the studied patients underwent re-resection, the survival outcomes measured, and the main conclusions with respect to the significant prognostic factors for re-resection of glioblastoma for each study included in the systematic review (*n* = 30). Abbreviations used in the table are defined as follows: first re-resection (*1RR)*, initial resection (*IR)*, time to re-resection/recurrence (*TTR)*, O6-methylguanine-DNA methyltransferase (*MGMT)*, glioblastoma (*GBM)*, Karnofsky Performance Status (*KPS)*, extent of resection (*EOR)*, gross total resection (*GTR)*, neutrophil–lymphocyte ratio (*NLR)*, overall survival (*OS)*, progression-free survival (*PFS)*

### Adjuvant therapy

Adjuvant therapy was only significantly associated with improved survival in eight studies [9, 11, 34, 46, 47, 50, 54, 57]. Four of these studies reported overall survival benefit (defined as survival following diagnosis of de novo GBM) [9, 11, 34, 50], while the other four reported improved survival after re-resection/recurrence of tumour [46, 47, 54, 57]. One study (Zanovello et al.) also found an association between adjuvant therapy following initial resection of primary GBM and increased survival; it also specified that adjuvant therapy after re-resection was only found to significantly improve survival where there was sub-total resection of recurrent GBM [54]. The remainder of studies found insignificant associations with both increased and reduced survival. Adjuvant treatments varied across studies ranging from systemic chemotherapeutic agents such as temozolomide (following Stupp protocol) to radiotherapy, stereotactic radiosurgery and gamma knife surgery (Table 1).

#### Meta-analysis

Despite significant findings in some of the included studies, on meta-analysis we could not demonstrate a significant survival benefit with studies reporting Cox proportional HR data, with high heterogeneity. Chemotherapy, the most commonly studied adjuvant therapy, did not show significant association with improved survival, with a pooled HR of 0.69 (95% CI 0.33–1.45, *p* = 0.33) (I^2^ = 81%, *p* < 0.01). Radiotherapy also did not demonstrate any benefit witha pooled HR of 0.62 (95% CI 0.15–2.48, *p* = 0.50) (I^2^ = 88%, *p* < 0.01)).Combined chemoradiotherapy, too, was not significant (HR 0.65, 95% CI 0.37–1.14, *p* = 0.13) (I^2^ = 49%, *p* = 0.16). (Supplementary Digital Content: Fig. 2).

**Fig. 2:**
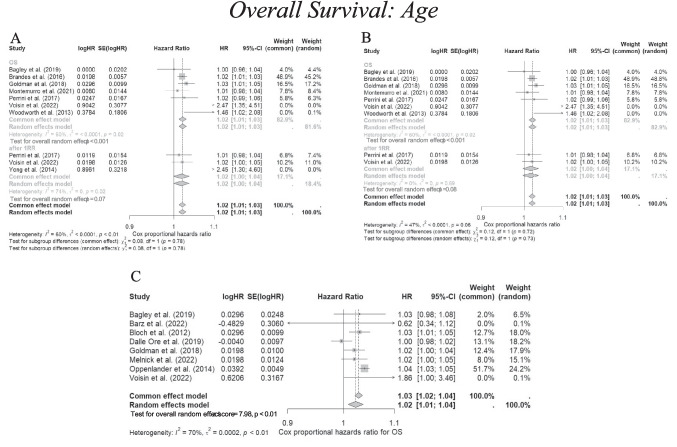
**A** A forest plot indicating the pooled univariate cox proportional hazard ratio representing the association between older age at the point of re-resection and overall survival. **B** The same forest plot excluding Yong et al., which was found to have significant risk of bias using ROBINS-I. **C** A forest plot indicating the multivariate cox proportional hazard ratio representing the association between older age at the point of re-resection and overall survival. A hazard ratio < 1.00 indicates association with increased survival, whereas a hazard ratio > 1.00 indicates association with worse survival. The weighting of each study is derived from the inverse of the variance of each study’s estimate hazard ratio. The size of the grey square is inversely proportional to the standard error, and the straight line indicates the 95% confidence intervals, which are shown in the square brackets. The diamonds indicate the overall pooled hazard ratio, and the random effects model is reported as the outcome. Heterogeneity is indicated by the I^2^ and tau.^2^ values. P value < 0.05 is deemed significant. Furthermore, for every study the following are displayed: study author with publication date (“Study”), HR, log(HR), the standard error of logHR (SElog(HR)), 95% confidence intervals, and the weighting of each study in percentage (%). A significant pooled hazard ratio for older age was found in both univariate (1.02) and multivariate (1.02) forest plot analyses but shows only a negligible association between older age and worse survival. Heterogeneity was statistically significant (*p* < 0.01)

### Age

Eight studies concluded that there was significant negative association between age and overall survival across univariate and multivariate analyses [11, 20, 29, 36, 38, 51, 53, 57].

#### Meta-analysis

While older age was associated with worse survival in individual studies, the effect size was small. The pooled univariate HR was 1.02 (95% CI 1.01–1.03, *p* < 0.001) (I^2^ = 60%, *p* = 0.02), and the multivariate HR was 1.02 (95% CI 1.01–1.04, *p* < 0.01) (I^2^ = 70%, *p* < 0.01). These findings suggest that age alone is not a strong independent prognostic factor. (Fig. 2A–C).

### Extent of resection

In our study, the definition of Gross Total Resection (GTR) encompassed both complete resection and near-total resection of the tumour and was found to significantly improve overall survival when compared with sub-total re-resection (STR) in 10 studies [3, 15, 21, 29, 34, 36, 48, 51, 54, 57], with four studies concluding there was no significant benefit.

#### Meta-analysis

**Fig. 3:**
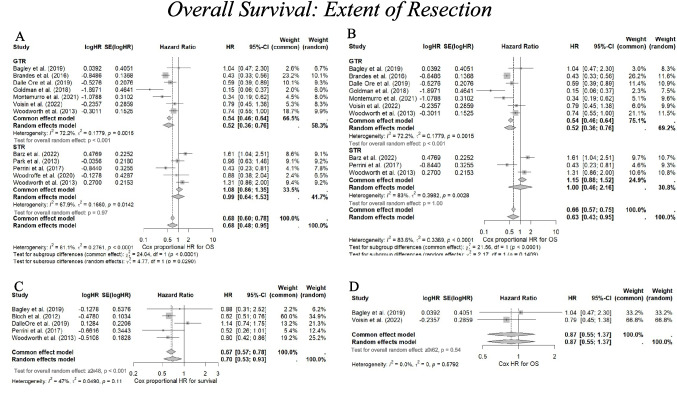
**A** A forest plot indicating the univariate cox proportional hazard ratio representing the association between extent of resection (EOR) and overall survival. EOR is split into subgroups of gross total resection (GTR), here defined as encompassing both total resection of the recurrent tumour and near-total resection, and subtotal resection (STR). **B** A forest plot indicating the univariate cox proportional hazard ratio representing the association between extent of resection (EOR) and overall survival (OS), this time excluding studies Park et al. and Woodroffe et al. which scored a high risk of bias using the ROBINS-I tool. **C** A forest plot indicating the multivariate cox proportional hazard ratio representing the association between gross total resection (GTR) and overall survival. **D** A forest plot indicating the univariate cox proportional hazard ratio representing the association between EOR and OS in studies only including IDH-wildtype glioblastoma patients. A hazard ratio < 1.00 indicates association with increased survival, whereas a hazard ratio > 1.00 indicates association with worse survival. The weighting of each study is derived from the inverse of the variance of each study’s estimate hazard ratio. The size of the grey square is inversely proportional to the standard error, and the straight line indicates the 95% confidence intervals, which are shown in the square brackets. The diamonds indicate the overall pooled hazard ratio, and the random effects model is reported as the outcome. Heterogeneity is indicated by the I^2^ and tau.^2^ values. *P* value < 0.05 is deemed significant. Furthermore, for every study the following are displayed: study author with publication date (“Study”), HR, log(HR), the standard error of logHR (SElog(HR)), 95% confidence intervals, and the weighting of each study in percentage (%). A significant pooled hazard ratio for GTR was found in both univariate (0.67) and multivariate (0.70) forest plot analyses, but not with STR. Heterogeneity was statistically significant (*p* < 0.01) in the univariate forest plot analysis, but not with forest plot of multivariate hazard ratios (*p* = 0.11)

GTR significantly improved survival, with a pooled univariate HR of 0.52 (95% CI 0.36–0.76, *p* < 0.001) (I^2^ = 68%, *p* = 0.01) and a multivariate HR of 0.70 (95% CI 0.53–0.93, *p* = 0.01) (I^2^ = 47%, *p* = 0.11,). Subtotal resection (STR) did not show a survival benefit (HR 0.99, 95% CI 0.64–1.53, *p* = 0.971). (Fig. 3A, C).

### Karnofsky performance scale

The Karnofsky Performance Scale (KPS) is the most used performance score for glioblastoma in clinical practice, ranging from 10 to 100 [41, 49]. This scale was universally reported by the studies included in this review. Studies differed in the threshold preoperative KPS status they used to compare survival outcome in patients that scored above and those that scored below; most used 70, the lowest score at which a patient is ambulatory and completely independent in their care needs [2], with the next most common being 80. Importantly, given most studies were retrospective, many of the patient cohorts selected to undergo re-resection were done so partly based on performance status and this therefore would have incurred selection bias. Despite this, five studies concluded that preoperative KPS score below a designated threshold was significantly associated with poor survival [3, 20, 29, 46, 50].

#### Meta-analysis

**Fig. 4:**
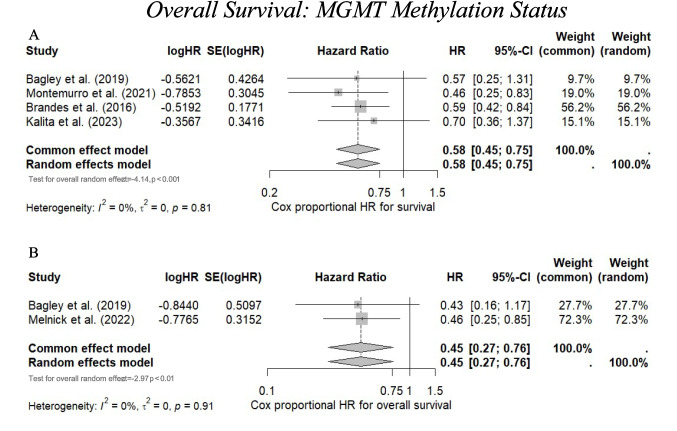
**A** A forest plot indicating the univariate cox proportional hazard ratio representing the association between methylated MGMT promoter status and overall survival (OS). **B** A forest plot indicating the multivariate cox proportional hazard ratio representing the association between methylated MGMT promoter status and OS. A hazard ratio < 1.00 indicates association with increased survival, whereas a hazard ratio > 1.00 indicates association with worse survival. The weighting of each study is derived from the inverse of the variance of each study’s estimate hazard ratio. The size of the grey square is inversely proportional to the standard error, and the straight line indicates the 95% confidence intervals, which are shown in the square brackets. The diamonds indicate the overall pooled hazard ratio, and the random effects model is reported as the outcome. Heterogeneity is indicated by the I^2^ and tau.^2^ values. *P* value < 0.05 is deemed significant. Furthermore, for every study the following are displayed: study author with publication date (“Study”), HR, log(HR), the standard error of logHR (SElog(HR)), 95% confidence intervals, and the weighting of each study in percentage (%). A significant pooled hazard ratio for older age was found in both univariate (0.58) and multivariate (0.45) forest plot analyses, showing improved association with survival for methylated MGMT promoter status. Heterogeneity was not statistically significant (*p* = 0.91)

A preoperative KPS score of < 70 was significantly associated with poorer survival. The pooled multivariate HR was 2.25 (95% CI 1.59–3.19, *p* < 0.001) (I^2^ = 0%, *p* = 0.41), suggesting that patients with better preoperative performance benefit more from re-resection. Univariate analyses and studies using a threshold of 80 did not find a significant association. (Supplementary Digital Content: Fig. 3A–B, Fig. 4A-B).

### Promoter methylation

MGMT promoter methylation has been widely accepted as a predictive biomarker for prognosis in glioblastoma patients undergoing treatment with alkylating agents such as temozolomide [24]. Most studies lacked survival outcome data for MGMT promoter methylation, with only three studies concluding it is significantly associated with increased survival [7, 34, 51].

#### Meta-analysis

Methylated MGMT promoter status, evaluated at the time of recurrence, was significantly associated with improved survival. The pooled multivariate HR was 0.45 (95% CI 0.27–0.76, *p* < 0.01) (I^2^ = 0%, *p* = 0.91), and the pooled univariate HR was 0.58 (95% CI 0.45–0.75, *p* < 0.001) (I^2^ = 0%, *p* = 0.81), both with low heterogeneity (*p* > 0.8). (Fig. 4A–B).

### Time to re-resection/recurrence

Time to re-resection and time to recurrence (TTR) from the point of first resection were treated as the same in this systematic review, owing to the paucity of studies reporting on each when studied separately. Here it was assumed that re-resection took place soon after first recurrence and that the interval in between had no effect on survival outcome. Studies compared TTR differently e.g., some investigating effect of survival for patients with a TTR of greater than six months with patients that had a shorter period [17], while others used the median TTR as the threshold. Goldman et al., used a different approach to show that while re-resection is significantly associated with increased survival when not accounting for timing, this effect is not observed when TTR is considered [26, 36]. Six studies concluded that a longer TTR is associated with increased overall survival or survival after re-resection [9, 17, 27, 29, 36, 47].

#### Meta-analysis

**Fig. 5:**
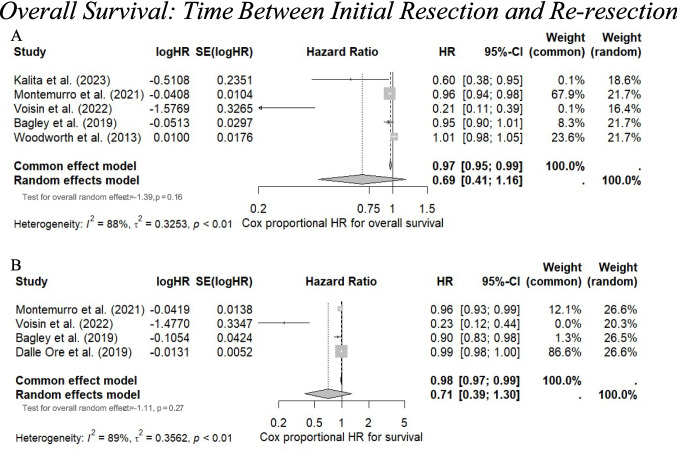
**A** A forest plot indicating the univariate cox proportional hazard ratio representing the association between a longer time between initial resection and re-resection/recurrence (TTR) and overall survival. **B** A forest plot indicating the multivariate cox proportional hazard ratio representing the association between a longer time between initial resection and re-resection/recurrence (TTR) and overall survival. A hazard ratio < 1.00 indicates association with increased survival, whereas a hazard ratio > 1.00 indicates association with worse survival. The weighting of each study is derived from the inverse of the variance of each study’s estimate hazard ratio. The size of the grey square is inversely proportional to the standard error, and the straight line indicates the 95% confidence intervals, which are shown in the square brackets. The diamonds indicate the overall pooled hazard ratio, and the random effects model is reported as the outcome. Heterogeneity is indicated by the I^2^ and tau.^2^ values. *P* value < 0.05 is deemed significant. Furthermore, for every study the following are displayed: study author with publication date (“Study”), HR, log(HR), the standard error of logHR (SElog(HR)), 95% confidence intervals, and the weighting of each study in percentage (%). An insignificant pooled hazard ratio for older age was found in both univariate and multivariate forest plot analyses. Heterogeneity was statistically significant (*p* < 0.01)

Despite six studies reporting a longer TTR being associated with improved survival, the meta-analysis revealed no significant association. The pooled univariate HR was 0.69 (95% CI 0.41–1.16, *p* = 0.16) (I^2^ = 88%, *p* < 0.01), and the multivariate HR was 0.71 (95% CI 0.39–1.30, *p* = 0.27) (I^2^ = 89%, *p* < 0.01). (Fig. 5A–B).

### IDH-wildtype only studies

Of the included studies, 22 were published prior to the 2021 WHO classification of glioblastoma. Only 11 studies reported IDH mutation status. Among these, four exclusively included patients with IDH-wildtype glioblastoma [3, 11, 17, 27], while the remaining seven reported predominantly IDH-wildtype cohorts with only a small proportion of IDH-mutant cases; the highest proportion was 12% in the study by Kalita et al. [13]. Of the four studies that included exclusively IDH-wildtype patients, three [3, 17, 27] reported Cox proportional hazard ratio data and could therefore be incorporated into the meta-analysis. Between these three studies, hazard ratios (HRs) were reported for age, GTR and TTR. For age, two studies provided univariate HRs, with a pooled HR of 1.49 (95% CI: 0.62–3.60, *p* = 0.37) (I^2^ = 88%, *p* < 0.01). All three provided multivariate HRs for age, with a pooled HR of 1.05 (95% CI: 0.62–1.78, *p* = 0.86) (I^2^ = 68%, *p* = 0.04). Two studies reported HRs for extent of resection (EOR), with a pooled univariate HR of 0.87 (95% CI: 0.55–1.37, *p* = 0.54) (I^2^ = 0%, *p* = 0.58). Two studies reported HRs for TTR in both univariate and multivariate analyses, with a pooled univariate HR of 0.46 (95% CI: 0.10–2.04, *p* = 0.31) (I^2^ = 95%, *p* < 0.001), and a pooled multivariate HR of 0.47 (95% CI: 0.12–1.81, *p* = 0.27) (I^2^ = 94%, *p* < 0.001). Forest plots restricted to wildtype-exclusive studies are presented for EOR (Fig. 3D), age (Supplementary Digital Content: Fig. 5A–B) and TTR (Supplementary Digital Content: Fig. 6A–B).

## Summary of results

### Significant positive predictors of survival

Gross Total Resection significantly improved survival compared to Subtotal Resection, with pooled univariate and multivariate HRs of 0.52 (95% CI: 0.36–0.76, *p* < 0.001) and 0.70 (95% CI: 0.53–0.93, *p* = 0.01), respectively. Methylated MGMT promoter status was associated with improved survival, with multivariate and univariate HRs of 0.45 (95% CI: 0.27–0.76, *p* < 0.01)and 0.58 (95% CI: 0.45–0.75, *p* < 0.001).

### Significant negative predictors of survival

Older age was significantly associated with worse survival, with pooled univariate and multivariate HRs of 1.02 (95% CI: 1.01–1.03, *p* < 0.001) and 1.02 (95% CI: 1.01–1.04, *p* < 0.01), respectively. Preoperative KPS scores ≤ 70 predicted worse outcomes (HR = 2.25, 95% CI: 1.59–3.19, *p* < 0.001).

### Non-significant predictors of survival

Chemotherapy was not associated with a significant survival benefit (HR = 0.69, 95% CI: 0.33–1.45, *p* = 0.33), and neither was radiotherapy (HR = 0.62, 95% CI: 0.15–2.48, *p* = 0.50). Combined chemoradiotherapy (HR = 0.65, 95% CI: 0.37–1.14, *p* = 0.13) and time to re-resection or recurrence (univariate HR = 0.69, 95% CI: 0.41–1.16, *p* = 0.16; multivariate HR = 0.71, 95% CI: 0.39–1.30, *p* = 0.27) did not show statistically significant associations with survival.

## Discussion

This meta-analysis, encompassing 18 studies with a pooled sample size of 1,741 patients, provides robust evidence for the prognostic value of key factors influencing survival after re-resection of glioblastoma. Among these, Gross Total Resection (GTR) and MGMT promoter methylation emerged as the most significant predictors of improved survival. The pooled multivariate hazard ratios for GTR (HR = 0.70, 95% CI: 0.53–0.93) and methylated MGMT promoter status (HR = 0.45, 95% CI: 0.27–0.76) highlight their critical roles in the management of recurrent glioblastoma. These findings underscore the importance of personalized and aggressive treatment strategies in this challenging patient population.

### Gross total resection

Maximal surgical resection at recurrence demonstrated the greatest survival benefit among the analyzed predictors, consistent with previous evidence for initial resections. GTR provides the opportunity to minimize residual tumour burden, which is strongly linked to tumour progression and poorer outcomes. Importantly, our analysis revealed that even when initial resection was subtotal, achieving GTR during re-resection significantly improved survival. This underscores the utility of adopting a proactive surgical approach whenever feasible, especially in patients with preserved functional status and manageable tumour location.

However, achieving GTR in recurrent glioblastoma remains challenging, particularly in cases involving eloquent brain regions or subventricular zone involvement [4, 28, 39]. The integration of advanced intraoperative tools, such as 5-aminolevulinic acid (5-ALA) fluorescence-guided resection and diffusion tensor imaging (DTI), has shown promise in overcoming these limitations [3, 14, 37]. For example, Woo et al. demonstrated that 5-ALA guidance improved the likelihood of achieving GTR and enhanced survival, though caution is required to avoid over-resection of normal tissues, which could lead to neurological deficits [37]. Future studies should evaluate the systematic application of these adjuncts in improving surgical outcomes at recurrence.

### MGMT promoter methylation

Methylation of the MGMT promoter is a well-established biomarker for predicting the efficacy of alkylating agents such as temozolomide in glioblastoma. Our findings confirm its strong association with improved survival following re-resection, with a pooled HR of 0.45 (95% CI: 0.27–0.76) [7, 34, 51]. This suggests that patients with methylated MGMT promoter status derive substantial benefits from re-resection when paired with adjuvant alkylating chemotherapy. Given the potential predictive power of this biomarker, routine testing of MGMT promoter methylation in recurrent glioblastoma is warranted to guide therapeutic decision-making.

### Additional significant predictors

In addition to GTR and MGMT promoter methylation, preoperative Karnofsky Performance Scale (KPS) scores was also significantly associated with survival in multivariate analyses. Preoperative KPS, a widely used functional score, demonstrated that patients with scores < 70 were less likely to benefit from re-resection (HR = 2.25, 95% CI: 1.59–3.19) [3, 20, 29, 46, 50]. This highlights the importance of careful patient selection, as those with better baseline performance status are more likely to tolerate surgery and subsequent adjuvant treatments.

### Non-significant predictors

Notably, some factors traditionally considered relevant for survival in glioblastoma failed to show consistent or significant associations in this analysis. Time to re-resection or recurrence, while hypothesized to reflect tumour biology and aggressiveness, did not yield a survival benefit in our meta-analysis (HR = 0.69, 95% CI: 0.41–1.16 for univariate analyses) [9, 17, 27, 29, 36, 47]. The significant heterogeneity (I^2^ = 88%, *p* < 0.01) suggests that differences in study design and reporting may have influenced these findings. It is also worth noting that early detection and intervention at recurrence could be a confounder, as it might allow for more complete resections and consequently improved overall outcomes. Similarly, adjuvant chemotherapy (HR = 0.69, 95% CI: 0.33–1.45), radiotherapy (HR = 0.62, 95% CI: 0.15–2.48), and combined adjuvant 7chemoradiotherapy (HR = 0.65, 95% CI: 0.37–1.14), though theoretically advantageous, did not show a survival benefit [9, 11, 34, 46, 47, 50, 54, 57]. The heterogeneity in how adjuvant therapies and medications were defined and reported across studies prevented the meaningful separation of treatment modalities in the analysis, which may have obscured potential differences in their individual effects. Although we found no prognostic effect of adjuvant therapy after re-resection, Karschnia et al. reported that absence of post-operative therapy at recurrence was significantly associated with worse survival [8]. These results highlight the need for more detailed subgroup analyses to elucidate the specific contexts in which these interventions may be effective. Age, though modestly associated with worse survival in individual studies, also failed to emerge as a strong prognostic factor in our pooled analysis, with a small effect size (HR = 1.02, 95% CI: 1.01–1.03) [11, 20, 29, 36, 38, 51, 53, 57]. This suggests that chronological age alone should not preclude aggressive treatment approaches, especially in functionally robust patients.

#### Interpretation of MGMT and adjuvant chemotherapy

The prognostic implications of MGMT promoter methylation and adjuvant chemotherapy remain an area of ongoing uncertainty. While our pooled analysis confirmed MGMT methylation as a significant predictor of prolonged survival, adjuvant chemotherapy did not independently correlate with improved outcome in the aggregated dataset. This likely reflects both clinical selection effects and the limitations of the available evidence, as most included studies did not stratify post-resection chemotherapy regimens by MGMT status or provide patient-level covariate data allowing adjustment for confounding. Consequently, this meta-analysis could not perform a fully adjusted multivariate regression to jointly evaluate MGMT methylation, chemotherapy, and other clinical parameters. Within these constraints, MGMT methylation at recurrence should be interpreted primarily as a prognostic marker rather than a predictive biomarker for temozolomide efficacy, although a differential treatment response in MGMT-methylated patients cannot be excluded. Future individual-patient data (IPD) meta-analyses will be essential to disentangle these effects and define whether MGMT-methylated patients derive disproportionate benefit from temozolomide rechallenge after reoperation.

## Limitations

The present findings must also be interpreted in the context of the methodological variability across the included studies. Definitions of gross total resection were inconsistent (e.g., thresholds of > 90% vs. > 95% resection of contrast-enhancing tumour), as were criteria for eloquent region involvement and the temporal reference points for survival metrics (time to recurrence vs. time to reoperation). Only one study (Woodroffe et al.) assessed the prognostic relevance of the extent of resection beyond the contrast-enhancing lesion. They found no significant association between resection of FLAIR hyperintensity or the ratio of enhancing to non-enhancing tumour volume and overall survival. This heterogeneity highlights the need for standardized radiological and clinical definitions to enable more robust quantitative synthesis and improve comparability across future glioblastoma re-resection studies. Similarly, the anatomical site of the primary tumour was variably reported and often lacked sufficient granularity to allow for systematic comparison across studies. As a result, potential location-specific survival effects could not be quantitatively evaluated, underscoring the need for uniform reporting of anatomical parameters in future research.

Despite the insights provided by this meta-analysis, several limitations warrant consideration. The included studies were predominantly retrospective and exhibited significant heterogeneity, reflecting variability in study design, patient selection, and outcome reporting. Additionally, non-survival metrics such as quality of life and neurological morbidity were underreported, limiting the scope of this analysis. Few studies evaluated the impact of advanced surgical adjuncts, such as fluorescence guidance or intraoperative imaging, which could further refine the benefits of GTR. Most studies did not report IDH mutation status, and only four included exclusively IDH-wildtype patients. Sensitivity analyses restricted to these studies did not yield significant associations, likely due to the limited number of available datasets. As such, it remains uncertain whether the observed associations between survival and prognostic factors at re-resection can be generalized to IDH-wildtype glioblastoma alone. Nevertheless, among the 11 studies that did report IDH status, patient cohorts were predominantly IDH-wildtype, suggesting that the primary findings of this review are still largely reflective of this population, in line with the 2021 WHO classification.

Although our findings did not yield a statistically significant survival benefit from adjuvant therapies following recurrence, this does not preclude the potential impact of emerging targeted treatments. Individualised treatments such as BRAF mutation inhibitors may play a more pivotal role, particularly at recurrence, where molecular profiling through whole genome sequencing can uncover actionable mutations and guide personalized therapeutic strategies [35, 55]. Future studies should explore the influence of these molecular markers and integration of such precision approaches to better stratify patients and optimize outcomes.

## Conclusion

This systematic review and meta-analysis identified several key prognostic factors influencing survival following re-resection of glioblastoma. Significant positive predictors included Gross Total Resection (HR = 0.52, 95% CI: 0.36–0.74, *p* < 0.001), methylated MGMT promoter status (HR = 0.45, 95% CI: 0.27–0.76, *p* < 0.01), and preoperative KPS ≥ 70 (HR = 2.25, 95% CI: 1.59–3.19, *p* < 0.001). In contrast, older age was associated with poorer outcomes (HR = 1.02, 95% CI: 1.01–1.03, *p* < 0.001). However, time to re-resection and adjuvant chemotherapy, radiotherapy and combined chemoradiotherapy did not show significant associations with survival.

Overall, this meta-analysis reinforces the importance of Gross Total Resection and MGMT promoter methylation as pivotal predictors of survival in recurrent glioblastoma. Specifically, re-resection should be considered in patients with favourable performance status and tumour characteristics, including methylated MGMT promoter status, and where Gross Total Resection is feasible. Nonetheless, despite these findings, the significant heterogeneity among studies and retrospective nature of the data underscore the need for high-quality prospective trials to refine treatment paradigms for recurrent glioblastoma. These insights provide a foundation for future research aimed at optimizing outcomes for this challenging patient population.

## Supplementary information

Below is the link to the electronic supplementary material.

ESM 1 Supplementary Material 1 (DOCX 8.54)

## Data Availability

All relevant data supporting the findings of this study can be accessed within the Supplementary Digital Content attached to the article. Additionally, a comprehensive dataset used for the meta-analysis is freely available and can be retrieved from the public GitHub repository. To ensure transparency and replicability of the research, the repository includes both raw data and processed data utilized in the study. Please visit the following link for access: https://github.com/ManuelVBaby/Glioblastoma-re-resection-prognostic-factors-MA.git
