# Supplementary material for: Determinants of survival after re-resection for recurrent glioblastoma: a meta-analysis
Source: Acta Neurochir (Wien). 2026 Jan 13;168(1):11. doi: 10.1007/s00701-025-06755-6 (PMC12804209; doi:10.1007/s00701-025-06755-6)
Supplement: Supplementary file 1 — Supplementary Material 1 (DOCX 8.54) [file 701_2025_6755_MOESM1_ESM.docx]

**SUPPLEMENTAL DIGITAL CONTENT**

**Prognostic Factors Influencing Survival After**

**Re-Resection in Recurrent Glioblastoma:**

**A Meta-Analysis.**

Manuel V. Baby^1,2^

Rithvik M. Narendranath^1,2^  ; Symriti Kaur-Paneser^1,2^

Daniele S.C. Ramsay^1,2^ ;Hariharan Subbiah Ponniah^1,2^

Srikar Namireddy^1,2^  ; Ahmed Salih^1,2^ ; Ahkash Thavarajasingam^1,3^ ;

Daniel Scurtu^4^; Andreas Kramer^4^; Veit Stöcklein^5^; Darius Kalasauskas^5^; Dragan Jankovic^5^ ; Florian Ringel^5^;

Santhosh G. Thavarajasingam^1,5 *^

**INSTITUTION:**

1. Imperial Brain and Spine Initiative, Imperial College London, London, United Kingdom
2. Faculty of Medicine, Imperial College London, London, United Kingdom.
3. Faculty of Medicine, Medizinische Hochschule Hannover, Hannover, Germany.
4. Department of Neurosurgery, University Medical Center Mainz, Mainz, Germany
5. Department of Neurosurgery, LMU University Hospital, LMU Munich, Germany.

Table of Contents

[Supplementary Table 1: PRISMA Reporting Statement. 3](#_Toc207310055)

[Supplementary Table 2: Search strategy. 6](#_Toc207310056)

[Supplementary Table 3: Inclusion and Exclusion criteria. 7](#_Toc207310057)

[Supplementary Table 4: Table of all extracted variables 8](#_Toc207310058)

[Supplementary Table 5: Oxford Centre of Evidence-Based Medicine (OCEBM) Levels of Evidence. 9](#_Toc207310059)

[Supplementary Table 6: ROBINS-I Risk of Bias Scoring. 11](#_Toc207310060)

[Supplementary Table 7: The R code. 12](#_Toc207310061)

[Supplementary Figure 1: Traffic light plot of risk of bias analysis (ROBINS-I). 22](#_Toc207310062)

[Supplementary Figure 2: Forest plot showing univariate cox HR for adjuvant chemoradiotherapy. 23](#_Toc207310063)

[Supplementary Figure 3: Forest plots showing univariate and multivariate cox HR for KPS < 70. 25](#_Toc207310064)

[Supplementary Figure 4: Forest plots showing univariate and multivariate cox HR for KPS < 80. 27](#_Toc207310065)

[Supplementary Figure 5: Forest plots showing univariate and multivariate cox HR for Age including studies with recurrence in only IDH-wildtype glioblastoma. 29](#_Toc207310066)

[Supplementary Figure 6: Forest plots showing univariate and multivariate cox HR for time to recurrence/re-resection (TTR) including studies with recurrence in only IDH-wildtype glioblastoma. 32](#_Toc207310067)

[References 34](#_Toc207310068)

#

# **Supplementary Table 1:** PRISMA Reporting Statement.

| **Section and Topic** | **Item #** | **Checklist item** | **Location where item is reported** |
| --- | --- | --- | --- |
| **TITLE** | | |  |
| Title | 1 | Identify the report as a systematic review. | Page 1 |
| **ABSTRACT** | | |  |
| Abstract | 2 | See the PRISMA 2020 for Abstracts checklist. | Page 2 |
| **INTRODUCTION** | | |  |
| Rationale | 3 | Describe the rationale for the review in the context of existing knowledge. | Pages 2-3 |
| Objectives | 4 | Provide an explicit statement of the objective(s) or question(s) the review addresses. | Page 2-3 |
| **METHODS** | | |  |
| Eligibility criteria | 5 | Specify the inclusion and exclusion criteria for the review and how studies were grouped for the syntheses. | Supplementary Table 3 |
| Information sources | 6 | Specify all databases, registers, websites, organisations, reference lists and other sources searched or consulted to identify studies. Specify the date when each source was last searched or consulted. | Page 4 |
| Search strategy | 7 | Present the full search strategies for all databases, registers and websites, including any filters and limits used. | Supplementary Table 2 |
| Selection process | 8 | Specify the methods used to decide whether a study met the inclusion criteria of the review, including how many reviewers screened each record and each report retrieved, whether they worked independently, and if applicable, details of automation tools used in the process. | Page 4 |
| Data collection process | 9 | Specify the methods used to collect data from reports, including how many reviewers collected data from each report, whether they worked independently, any processes for obtaining or confirming data from study investigators, and if applicable, details of automation tools used in the process. | Page 4-5 |
| Data items | 10a | List and define all outcomes for which data were sought. Specify whether all results that were compatible with each outcome domain in each study were sought (e.g. for all measures, time points, analyses), and if not, the methods used to decide which results to collect. | Page 4-5 and Tables 1, 2 |
|  | 10b | List and define all other variables for which data were sought (e.g. participant and intervention characteristics, funding sources). Describe any assumptions made about any missing or unclear information. | Tables 1-2, Supplementary Table 4 |
| Study risk of bias assessment | 11 | Specify the methods used to assess risk of bias in the included studies, including details of the tool(s) used, how many reviewers assessed each study and whether they worked independently, and if applicable, details of automation tools used in the process. | Page 4, Figure 1B, Supplementary Table 6 and Supplementary Figure 1 |
| Effect measures | 12 | Specify for each outcome the effect measure(s) (e.g. risk ratio, mean difference) used in the synthesis or presentation of results. | Table 2, Supplementary Table 4 |
| Synthesis methods | 13a | Describe the processes used to decide which studies were eligible for each synthesis (e.g. tabulating the study intervention characteristics and comparing against the planned groups for each synthesis (item #5)). | Supplementary Table 4 |
|  | 13b | Describe any methods required to prepare the data for presentation or synthesis, such as handling of missing summary statistics, or data conversions. | Page 5 |
|  | 13c | Describe any methods used to tabulate or visually display results of individual studies and syntheses. | Page 5 |
|  | 13d | Describe any methods used to synthesize results and provide a rationale for the choice(s). If meta-analysis was performed, describe the model(s), method(s) to identify the presence and extent of statistical heterogeneity, and software package(s) used. | Page 5 |
|  | 13e | Describe any methods used to explore possible causes of heterogeneity among study results (e.g. subgroup analysis, meta-regression). | Page 5 |
|  | 13f | Describe any sensitivity analyses conducted to assess robustness of the synthesized results. | Not included |
| Reporting bias assessment | 14 | Describe any methods used to assess risk of bias due to missing results in a synthesis (arising from reporting biases). | Page 4 |
| Certainty assessment | 15 | Describe any methods used to assess certainty (or confidence) in the body of evidence for an outcome. | Supplementary Table 5 |
| **RESULTS** | | |  |
| Study selection | 16a | Describe the results of the search and selection process, from the number of records identified in the search to the number of studies included in the review, ideally using a flow diagram. | Figure 1A |
|  | 16b | Cite studies that might appear to meet the inclusion criteria, but which were excluded, and explain why they were excluded. | NA |
| Study characteristics | 17 | Cite each included study and present its characteristics. | Table 1 |
| Risk of bias in studies | 18 | Present assessments of risk of bias for each included study. | Figure 1B, Supplementary Table 6 and Supplementary Figure 1 |
| Results of individual studies | 19 | For all outcomes, present, for each study: (a) summary statistics for each group (where appropriate) and (b) an effect estimate and its precision (e.g. confidence/credible interval), ideally using structured tables or plots. | Figures 2-7, Supplementary Figure 2 |
| Results of syntheses | 20a | For each synthesis, briefly summarise the characteristics and risk of bias among contributing studies. | Table 1, Figure 1B, Supplementary Table 6 and Supplementary Figure 1 |
|  | 20b | Present results of all statistical syntheses conducted. If meta-analysis was done, present for each the summary estimate and its precision (e.g. confidence/credible interval) and measures of statistical heterogeneity. If comparing groups, describe the direction of the effect. | Pages 5-9 , Figures 2-7, Supplementary Figure 2 |
|  | 20c | Present results of all investigations of possible causes of heterogeneity among study results. | Page 13 |
|  | 20d | Present results of all sensitivity analyses conducted to assess the robustness of the synthesized results. | NA |
| Reporting biases | 21 | Present assessments of risk of bias due to missing results (arising from reporting biases) for each synthesis assessed. | Supplementary Figures 1 and 2 |
| Certainty of evidence | 22 | Present assessments of certainty (or confidence) in the body of evidence for each outcome assessed. | Supplementary Table 5 |
| **DISCUSSION** | | |  |
| Discussion | 23a | Provide a general interpretation of the results in the context of other evidence. | Page 9-11 |
|  | 23b | Discuss any limitations of the evidence included in the review. | Page 10-11 |
|  | 23c | Discuss any limitations of the review processes used. | Page 11 |
|  | 23d | Discuss implications of the results for practice, policy, and future research. | Page 9-12 |
| **OTHER INFORMATION** | | |  |
| Registration and protocol | 24a | Provide registration information for the review, including register name and registration number, or state that the review was not registered. | Page 4 |
|  | 24b | Indicate where the review protocol can be accessed, or state that a protocol was not prepared. | Page 4 |
|  | 24c | Describe and explain any amendments to information provided at registration or in the protocol. | NA |
| Support | 25 | Describe sources of financial or non-financial support for the review, and the role of the funders or sponsors in the review. | Title page/designated section in paper |
| Competing interests | 26 | Declare any competing interests of review authors. | Title page/designated section in paper |
| Availability of data, code and other materials | 27 | Report which of the following are publicly available and where they can be found: template data collection forms; data extracted from included studies; data used for all analyses; analytic code; any other materials used in the review. | Supplementary Table 7 |

This table shows the 27-point checklist furnished by the Preferred Reporting Items for Systematic Reviews and Meta-Analyses (PRISMA) 2020 statement, addressing the individual sections in this systematic review and meta-analysis. [1]

# **Supplementary Table 2:** Search strategy.

| **Database** | **Search terms** | **Publication dates** | **Results (n)** |
| --- | --- | --- | --- |
| Ovid Medline  &  Embase | ((Glioblastoma* or GBM or grade IV astrocytoma* or grade-IV astrocytoma* or grade 4 astrocytoma* or grade-4 astrocytoma* or grade IV glioma* or grade-IV glioma* or grade 4 glioma* or grade-4 glioma* or IDH wildtype* or IDH wild-type* or IDH wild type) and (re-resect* or reresect* or repeat resect* or repeat operat* or re-operat* or reoperat* or repeat surgery or second surgery or second resection)).mp. [ mp=ti, ab, hw, tn, ot, dm, mf, dv, kf, fx, dq, bt, nm, ox, px, rx, an, ui, sy, ux, mx] | 1946 – 12/01/2024 | 1,721 |
| Scopus | ((Glioblastoma* OR GBM OR grade IV astrocytoma* OR grade-IV astrocytoma* OR grade 4 astrocytoma* OR grade-4 astrocytoma* OR grade IV glioma* OR grade-IV glioma* OR grade 4 glioma* OR grade-4 glioma* OR IDH wildtype* OR IDH wild-type* OR IDH wild type) AND (re-resect* OR reresect* OR repeat resect* OR repeat operat* OR re-operat* OR reoperat* OR repeat surgery OR second surgery OR second resection)) | 2016 – 12/01/2024 | 26 |
| Pubmed | ((Glioblastoma* or GBM or grade IV astrocytoma* or grade-IV astrocytoma* or grade 4 astrocytoma* or grade-4 astrocytoma* or grade IV glioma* or grade-IV glioma* or grade 4 glioma* or grade-4 glioma* or IDH wildtype* or IDH wild-type* or IDH wild type) and (re-resect* or reresect* or repeat resect* or repeat operat* or re-operat* or reoperat* or repeat surgery or second surgery or second resection)) | 1976- 12/01/2024 | 1,763 |

Supplementary Table 2 shows the search strategy performed on 13^th^ January 2024, outlining the respective databases, search terms, publication dates chosen as limiting factors, and number of results from each database.

# **Supplementary Table 3**: Inclusion and Exclusion criteria.

| **Inclusion criteria** | **Exclusion criteria** |
| --- | --- |
| - Published in the English language - Peer-reviewed journals - Adult human patients with recurrent glioblastoma - Studies where at least a subset of patients undergoing re-resection for recurrent glioblastoma. - Comparison of survival outcomes for re-resection of glioblastoma in the presence of a specified prognostic factor versus re-resection in the absence of the factor, with comparable quantitative outcome measures. | - All non-English languages. - Commentaries, case reports, narrative reviews, letters to editors, books. - Any animal of lab-based studies. - Studies on children or adolescents <18 years. - Studies only looking at initial resection of glioblastoma. |

Supplementary Table 3 shows the inclusion and exclusion criteria criteria used when filtering studies during abstract and full-text screening based off search results (found in Supplementary Table 1).

# **Supplementary Table 4:** Table of all extracted variables

| **Extracted variables for qualitative synthesis** | **Extracted variables for quantitative synthesis** |
| --- | --- |
| - Study title, Authors, Follow-up period, Year of publication, Country, Study design, Regression model used, Sample size, Number of male patients, Number of female patients, Adjuvant therapy following Initial Resection, Neoadjuvant therapy/adjuvant therapy following re-resection, Number of patients with tumour recurrence, Number of patients with pseudo-progression/treatment-related changes, Prognostic factors studied, Main conclusions. | - Adjuvant therapy meta-analysis: author, adjuvant therapy, Cox proportional hazard ratio (HR), logHR, upper confidence interval (CI) of HR, lower CI of HR, log(upper CI), log(lower CI), variance of log HR, standard error (SE), p value - Age and MGMT univariate and multivariate meta-analyses: author, survival measure, HR, logHR, upper CI of HR, lower CI of HR, log(upper CI), log(lower CI), variance of log HR, standard error (SE), p value - Extent of Resection (EOR) univariate and multivariate meta-analysis (including without Park et al. and Woodroffe et al.): author, EOR, HR, logHR, upper CI of HR, lower CI of HR, log(upper CI), log(lower CI), variance of log HR, standard error (SE), p value - Karnofsky Performance Scale (KPS) univariate meta-analysis: author, KPS definition, HR, logHR, upper CI of HR, lower CI of HR, log(upper CI), log(lower CI), variance of log HR, standard error (SE), p value - KPS multivariate meta-analysis: author, KPS definition, risk of bias, HR, logHR, upper CI of HR, lower CI of HR, log(upper CI), log(lower CI), variance of log HR, standard error (SE), p value - Time to Recurrence/Re-resection (TTR) univariate and multivariate meta-analysis: author, time measure, HR, logHR, upper CI of HR, lower CI of HR, log(upper CI), log(lower CI), variance of log HR, standard error (SE), p value |

Supplementary Table 4 shows the extracted variables in the qualitative synthesis (systematic review) and quantitative synthesis (meta-analysis).

# **Supplementary Table 5:** Oxford Centre of Evidence-Based Medicine (OCEBM) Levels of Evidence.

| **Study number** | **Author, Year** | **Level of evidence** |
| --- | --- | --- |
| 1 | Bagley et al. (2019) | 3 |
| 2 | Barz et al. (2022) | 3 |
| 3 | Bloch et al. (2012) | 3 |
| 4 | Brandes et al. (2016) | 3 |
| 5 | Dalle Ore et al. (2019) | 3 |
| 6 | De Bonis et al. (2013) | 3 |
| 7 | Goldman et al. (2018) | 3 |
| 8 | Hennessy et al. (2022) | 3 |
| 9 | Kalita et al. (2023) | 3 |
| 10 | Mandl et al. (2008) | 3 |
| 11 | McNamara et al. (2014) | 3 |
| 12 | Melnick et al. (2022) | 3 |
| 13 | Montemurro et al. (2021) | 3 |
| 14 | Okita et al. (2012) | 3 |
| 15 | Oppenlander et al. (2014) | 3 |
| 16 | Park et al. (2013) | 3 |
| 17 | Patrizz et al. (2021) | 3 |
| 18 | Perrini et al. (2017) | 3 |
| 19 | Pessina et al. (2017) | 3 |
| 20 | Pinsker et al. (2001) | 3 |
| 21 | Quick et al. (2014) | 3 |
| 22 | Ringel et al. (2016) | 3 |
| 23 | Sonoda et al. (2014) | 3 |
| 24 | Suchorska et al. (2016) | 3 |
| 25 | Voisin et al. (2022) | 3 |
| 26 | Woo et al. (2023) | 3 |
| 27 | Woodroffe et al. (2020) | 3 |
| 28 | Woodworth et al. (2013) | 3 |
| 29 | Yong et al. (2014) | 3 |
| 30 | Zanovello et al. (2016) | 3 |

Supplementary Table 5 shows the results of the analysis of the strength/quality of scientific evidence of all included studies using the Oxford Centre of Evidence-Based Medicine (OCEBM) Levels of Evidence tool [2].

# **Supplementary Table 6:** ROBINS-I Risk of Bias Scoring.

| **Study** | **D1** | **D2** | **D3** | **D4** | **D5** | **D6** | **D7** | **Overall** |
| --- | --- | --- | --- | --- | --- | --- | --- | --- |
| Bagley 2019 | Moderate | Moderate | Low | Low | Low | Low | Moderate | Moderate |
| Barz 2022 | Moderate | Low | Low | Low | Low | Low | Low | Moderate |
| Bloch 2012 | Moderate | Moderate | Moderate | Low | Low | Low | Moderate | Moderate |
| Brandes 2016 | Moderate | Moderate | Low | Low | Low | Low | Moderate | Moderate |
| DalleOre 2019 | Moderate | Moderate | Low | Low | Low | Low | Moderate | Moderate |
| DeBonis 2013 | Moderate | Serious | Low | Moderate | Low | Low | Low | Serious |
| Goldman 2018 | Moderate | Low | Low | Low | Low | Low | Low | Moderate |
| Hennessy 2022 | Moderate | Moderate | Moderate | Low | Low | Low | Moderate | Moderate |
| Kalita 2023 | Moderate | Moderate | Low | Low | Moderate | Low | Moderate | Moderate |
| Mandl 2008 | Serious | Moderate | Low | Low | Low | Low | Low | Serious |
| McNamara 2014 | Moderate | Low | Low | Low | Low | Low | Low | Moderate |
| Melnick 2022 | Moderate | Moderate | Low | Low | Moderate | Low | Moderate | Moderate |
| Montemurro 2021 | Moderate | Low | Low | Low | Low | Low | Moderate | Moderate |
| Okita 2012 | Moderate | Low | Moderate | Low | Low | Low | Low | Moderate |
| Oppenlander 2014 | Moderate | Moderate | Moderate | Low | Low | Low | Moderate | Moderate |
| Park 2013 | Moderate | Moderate | Serious | Low | Low | Low | Moderate | Serious |
| Patrizz 2021 | Moderate | Low | Low | Moderate | Low | Low | Moderate | Moderate |
| Perrini 2017 | Moderate | Moderate | Low | Low | Low | Low | Low | Moderate |
| Pessina 2017 | Moderate | Low | Low | Low | Low | Low | Low | Moderate |
| Pinsker 2001 | Moderate | Moderate | Low | Low | Low | Moderate | Moderate | Moderate |
| Quick 2014 | Moderate | Low | Moderate | Low | Low | Low | Low | Moderate |
| Ringel 2016 | Moderate | Low | Moderate | Low | Low | Moderate | Low | Moderate |
| Sonoda 2014 | Moderate | Moderate | Low | Low | Low | Moderate | Low | Moderate |
| Suchorska 2016 | Moderate | Moderate | Low | Low | Moderate | Low | Low | Moderate |
| Voisin 2022 | Moderate | Moderate | Low | Low | Low | Low | Low | Moderate |
| Woo 2023 | Moderate | Moderate | Low | Low | Low | Low | Low | Moderate |
| Woodroffe 2020 | Moderate | Serious | Low | Low | Moderate | Low | Low | Serious |
| Woodworth 2013 | Moderate | Low | Low | Low | Low | Low | Moderate | Moderate |
| Yong 2014 | Moderate | Moderate | Low | Low | Low | Moderate | Serious | Serious |
| Zanovello 2016 | Moderate | Low | Low | Low | Low | Low | Low | Moderate |

Supplementary Table 6 shows the results of the risk of bias analysis of all included studies using the ROBINS-I tool [3]: All studies were non-randomised, and found to have at least a moderate risk of bias overall because of their retrospective nature and significant risk of confounding and bias the selection criteria of patients for re-resection based on variable such as age and pre-operative KPS status, which were investigated as prognostic factors under this review. De Bonis et al. [4], Mandl et al. [5], Park et al. [6], Woodroffe et al. [7], and Yong et al. [8], were found to have serious risk of bias. De Bonis et al. [4] and Woodroffe et al. [7] were found to have a serious risk of bias in the selection of participants in the study. Mandl et al. [5] was found to have a serious risk of bias due to confounding. Park et al. [6] had a serious risk of bias due to bias in classification of interventions, and Yong et al. [8] due selection of the reported results.

# **Supplementary Table 7:** The R code.

>

>

> # Install packages

> install.packages("meta")

> install.packages("metafor")

> install.packages("rmeta")

> install.packages("readxl")

> library("meta")

> library("rmeta")

> library("readxl")

>

> #Forest plot for Adjuvant therapy

>

> getwd()

> list.files(pattern="Adjuvant_Rx_HR_univariate")

> AdjuvantRx <- read_excel("Adjuvant_Rx_HR_univariate.xlsx")

> View(AdjuvantRx)

> mg1 <- metagen(logHR, se, studlab=paste(Study), data=AdjuvantRx, sm="HR")

> print(mg1, digits=2)

> mg1 <- update(mg1, byvar=Adjuvant_therapy, print.byvar=FALSE)

> mg1

> forest(mg1, xlim=c(0.05, 5), xlab = "Cox proportional hazards ratio for OS")

> summary(mg1)

> print(mg3, digits=2)

> mg3$bylab

> data.frame(

+ Subgroup = mg1$bylab,

+ HR = exp(mg1$TE.random.w),

+ Lower = exp(mg1$lower.random.w),

+ Upper = exp(mg1$upper.random.w),

+ P_value = mg1$pval.random.w

)

>

>

> # Age multivariate plot

> list.files(pattern="Age_HR_multivariate")

> Agemulti <- read_excel("Age_HR_multivariate.xlsx")

> mg2 <- metagen(logHR, se, studlab=paste(Study), data=Agemulti, sm="HR")

> print(mg2, digits=2)

> forest(mg2, xlim=c(0.9, 1.1), xlab="Cox proportional hazards ratio for OS")

>

>

>

> # Age univariate plot

> list.files=(pattern="Age_HR_univariate")

> Ageuni <- read_excel("Age_HR_univariate.xlsx")

> mg3 <- metagen(logHR, se, studlab=paste(Study), data=Ageuni, sm="HR")

> print(mg3, digits=2)

> mg3 <- update(mg3, byvar=Survival_measure, print.byvar=FALSE)

> mg3

> forest(mg3, xlim=c(0.9, 1.1) , xlab="Cox proportional hazards ratio")

>

>

>

>

> # EOR multivariate plot

> list.files=(pattern="EOR_HR_multivariate_analysis")

> list.files=(pattern="EOR_HR_multivariate_analysis")

> list.files(pattern="EOR_HR_multivariate_analysis")

> EORmulti <- read_excel("EOR_HR_multivariate_analysis.xlsx")

> mg4 <- metagen(logHR, se, studlab = paste(Study), data=EORmulti, sm="HR")

> forest(mg4, xlim=c(0.2, 3) , xlab="Cox proportional HR for survival")

>

>

>

>

> # EOR univariate plot

> list.files(pattern="EOR_HR_univariate_analysis")

> EORuni <- read_excel("EOR_HR_univariate_analysis.xlsx" )

> mg5 <- metagen(logHR, se, studlab=paste(Study), data=EORuni, sm="HR")

> mg5 <- update(mg5, byvar=EOR, print.byvar=FALSE)

> forest(mg5, xlab="Cox proportional hazards ratio for OS")

>

>

>

>

> # EOR univariate without Park and Woodroffe

> EORuni2 <- read_excel("EOR_HR_univariate_analysis_without_Park_and_Woodroffe.xlsx")

> mg6 <- metagen(logHR, se, studlab=paste(Study), data=EORuni2, sm="HR")

> mg6 <- update(mg6, byvar=EOR, print.byvar=FALSE)

> forest(mg6, xlab="Cox proportional hazards ratio for OS")

>

>

>

>

> # KPS 70 multivariate plot

> list.files(pattern="KPS70_HR_multivariate")

> KPS70multi <- read_excel("KPS70_HR_multivariate.xlsx")

> mg7 <- metagen(logHR, se, studlab=paste(Study), data=KPS70multi, sm="HR")

> mg7 <- update(mg7, byvar=Risk_of_bias, print.byvar=FALSE)

> forest(mg7, xlim=c(0.5, 8), xlab="Cox proportional HR for survival")

>

>

>

>

> # KPS 70 univariate plot

> list.files(pattern="KPS70_HR_univariate")

> KPS70uni <- read_excel("KPS70_HR_univariate.xlsx")

> mg8 <- metagen(logHR, se, studlab=paste(Study), data=KPS70uni, sm="HR")

> forest(mg8, xlab="Cox proportional HR for survival")

>

>

>

>

> # KPS 80 multi plot

> list.files(pattern="KPS80_HR_multivariate")

> KPS80multi <- read_excel("KPS80_HR_multivariate.xlsx")

> mg9 <- metagen(logHR, se, studlab=paste(Study), data=KPS80multi, sm="HR")

> forest(mg9, xlab="Cox proportional HR for survival")

> forest(mg9, xlim=c(0.2, 4) , xlab="Cox proportional HR for survival")

>

>

>

>

> # KPS 80 uni plot

> list.files(pattern="KPS80_HR_univariate")

> KPS80uni <- read_excel("KPS80_HR_univariate.xlsx")

> mg10 <- metagen(logHR, se, studlab=paste(Study), data=KPS80uni, sm="HR")

> forest(mg10, xlab="Cox proportional HR for survival")

> forest(mg10, xlim=c(0.1, 5) , xlab="Cox proportional HR for survival")

>

>

>

> # MGMT multi plot

> list.files(pattern="MGMT_HR_multivariate")

> MGMTmulti <- read_excel("MGMT_HR_multivariate.xlsx")

> mg11 <- metagen(logHR, se, studlab=paste(Study), data=MGMTmulti, sm="HR")

> forest(mg11, xlab="Cox proportional HR for survival")

> forest(mg10, xlim=c(0.1, 1.5) , xlab="Cox proportional HR for survival")

> forest(mg11, xlim=c(0.1, 1.5) , xlab="Cox proportional HR for survival")

>

>

>

>

> forest(mg11, xlim=c(0.1, 1.5) , xlab="Cox proportional HR for overall survival")

>

>

>

>

>

> # MGMT uni plot

> list.files(pattern="MGMT_HR_univariate")

> MGMTuni <- read_excel("MGMT_HR_univariate.xlsx")

> mg12 <- metagen(logHR, se, studlab=paste(Study), data=MGMTuni, sm="HR")

> forest(mg12, xlab="Cox proportional HR for survival")

> forest(mg12, xlim=c(0.2, 1.5) , xlab="Cox proportional HR for survival")

>

>

>

>

>

> # TTR multivariate plot

> list.files(pattern="TTR_HR_multivariate")

> TTRmulti <- read_excel("TTR_HR_multivariate.xlsx")

> mg13 <- metagen(logHR, se, studlab=paste(Study), data=TTRmulti, sm="HR")

> forest(mg13, xlab="Cox proportional HR for survival")

> forest(mg13, xlim=c(0.1, 1.5), xlab="Cox proportional HR for survival")

> forest(mg13, xlim=c(0.2, 1.5), xlab="Cox proportional HR for survival")

> forest(mg13, xlab="Cox proportional HR for survival")

>

>

>

> # TTR univariate plot

> list.files(pattern="TTR_HR_univariate")

> TTRuni <- read_excel("TTR_HR_univariate.xlsx")

> mg14 <- metagen(logHR, se, studlab=paste(Study), data=TTRuni, sm="HR")

> forest(mg14, xlab="Cox proportional HR for overall survival")

> forest(mg14, xlim=c(0.1, 1.5), xlab="Cox proportional HR for overall survival")

> forest(mg14, xlim=c(0.2, 1.5), xlab="Cox proportional HR for overall survival")

-------------------------------------------------------------------------------------------------------------

> # WORLD MAP PLOT

> # Sample data: publication countries with counts

> pub_countries <- data.frame(

+ country = c("Germany", "Italy", "USA", "South Korea", "Hong Kong", "Canada", "Australia", "Netherlands", "Japan", "Czech Republic", "Brazil", "Switzerland","Ireland" ),

+ count = c(4, 4, 12, 1, 1, 2, 1, 1, 2, 1, 1, 1, 1)

+ )

>

> # Get map data and merge with publication data

> world_map <- map_data("world")

> pub_countries <- pub_countries %>%

+ rename(region = country)

> world_map <- left_join(world_map, pub_countries, by = "region")

> world_map$count[is.na(world_map$count)] <- 0

>

> # Plot

> ggplot(data = world_map, aes(x = long, y = lat, group = group, fill = count)) +

+ geom_polygon(color = "white") +

+ scale_fill_gradient(low = "lightblue", high = "darkblue", na.value = "grey90") +

+ labs(title = "Choropleth Map of Publication Origins",

+ x = "Longitude",

+ y = "Latitude",

+ fill = "Number of Publications") +

+ theme_minimal()

# **
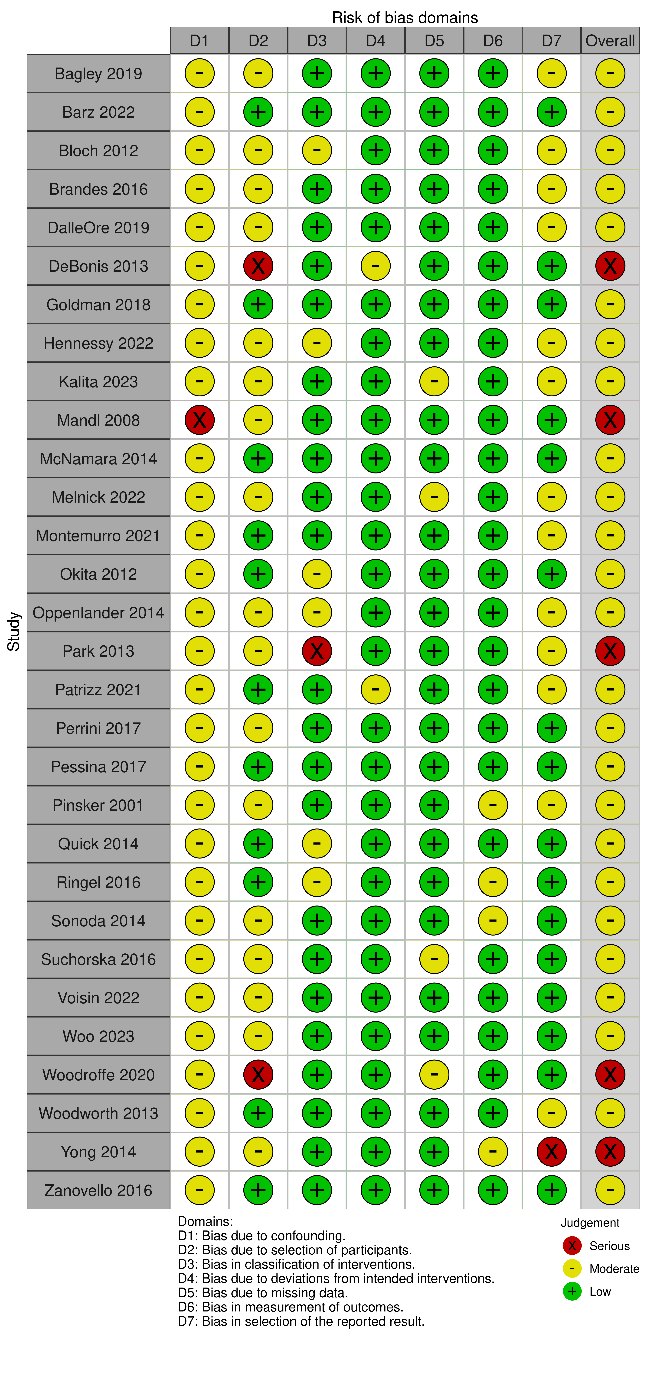
Supplementary Figure 1:** Traffic light plot of risk of bias analysis (ROBINS-I).

Supplementary Figure 1 shows a traffic light plot of the results of the risk of bias analysis of all included studies using the ROBINS-I tool [3]: The plot was created using the robvis web-app tool [9]. All studies were non-randomised and found to have a moderate risk of bias overall because of their retrospective nature and significant risk of confounding and bias the selection criteria of patients for re-resection based on variable such as age and pre-operative KPS status, which were investigated as prognostic factors under this review. De Bonis et al. [4], Mandl et al. [5], Park et al. [6], Woodroffe et al. [7], and Yong et al. [8], were found to have serious risk of bias. De Bonis et al. [4] and Woodroffe et al. [7] were found to have a serious risk of bias in the selection of participants in the study. Mandl et al. [5] was found to have a serious risk of bias due to confounding. Park et al. [6] had a serious risk of bias due to bias in classification of interventions, and Yong et al. [8] due selection of the reported results.

# **Supplementary Figure 2:** Forest plot showing univariate cox HR for adjuvant chemoradiotherapy.


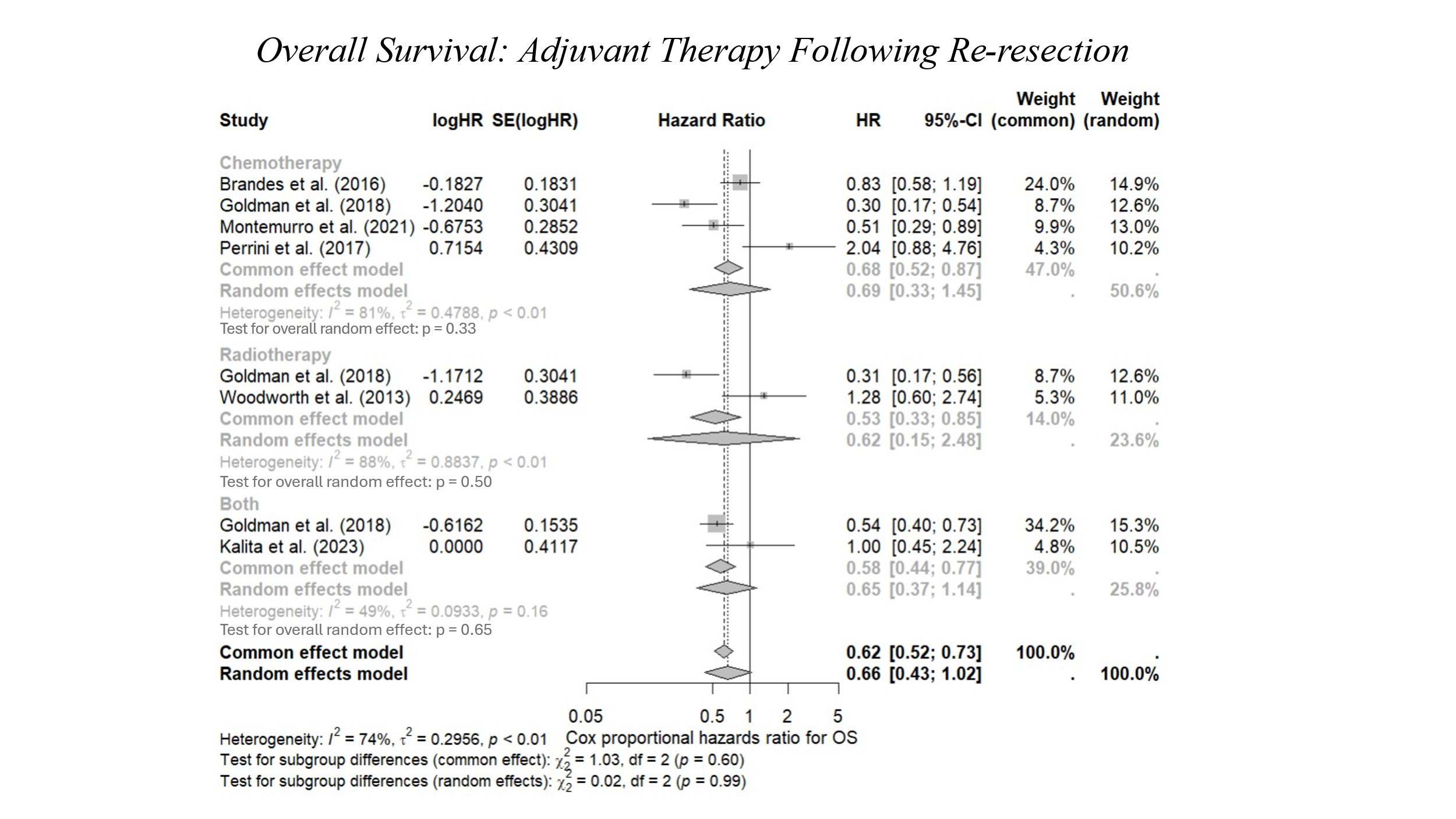


Supplementary Figure 2 shows a forest plot indicating the cox proportional hazard ratio (HR) representing the association between adjuvant chemotherapy and adjuvant radiotherapy following re-resection and overall survival (OS). A hazard ratio < 1.00 indicates association with increased survival, whereas a hazard ratio >1.00 indicates association with worse survival. The weighting of each study is derived from the inverse of the variance of each study’s estimate hazard ratio. The size of the grey square is inversely proportional to the standard error, and the straight line indicates the 95% confidence intervals, which are shown in the square brackets. The diamonds indicate the overall pooled hazard ratio, and the random effects model is reported as the outcome. Heterogeneity is indicated by the I^2^ and tau^2^ values. P value <0.05 is deemed significant. Furthermore, for every study the following are displayed: study author with publication date (“Study”), HR, log(HR), the standard error of logHR (SElog(HR)), 95% confidence intervals, and the weighting of each study in percentage (%). An insignificant pooled hazard ratio with survival was yielded for both chemotherapy and radiotherapy in this analysis.

# **Supplementary Figure 3:** Forest plots showing univariate and multivariate cox HR for KPS < 70.


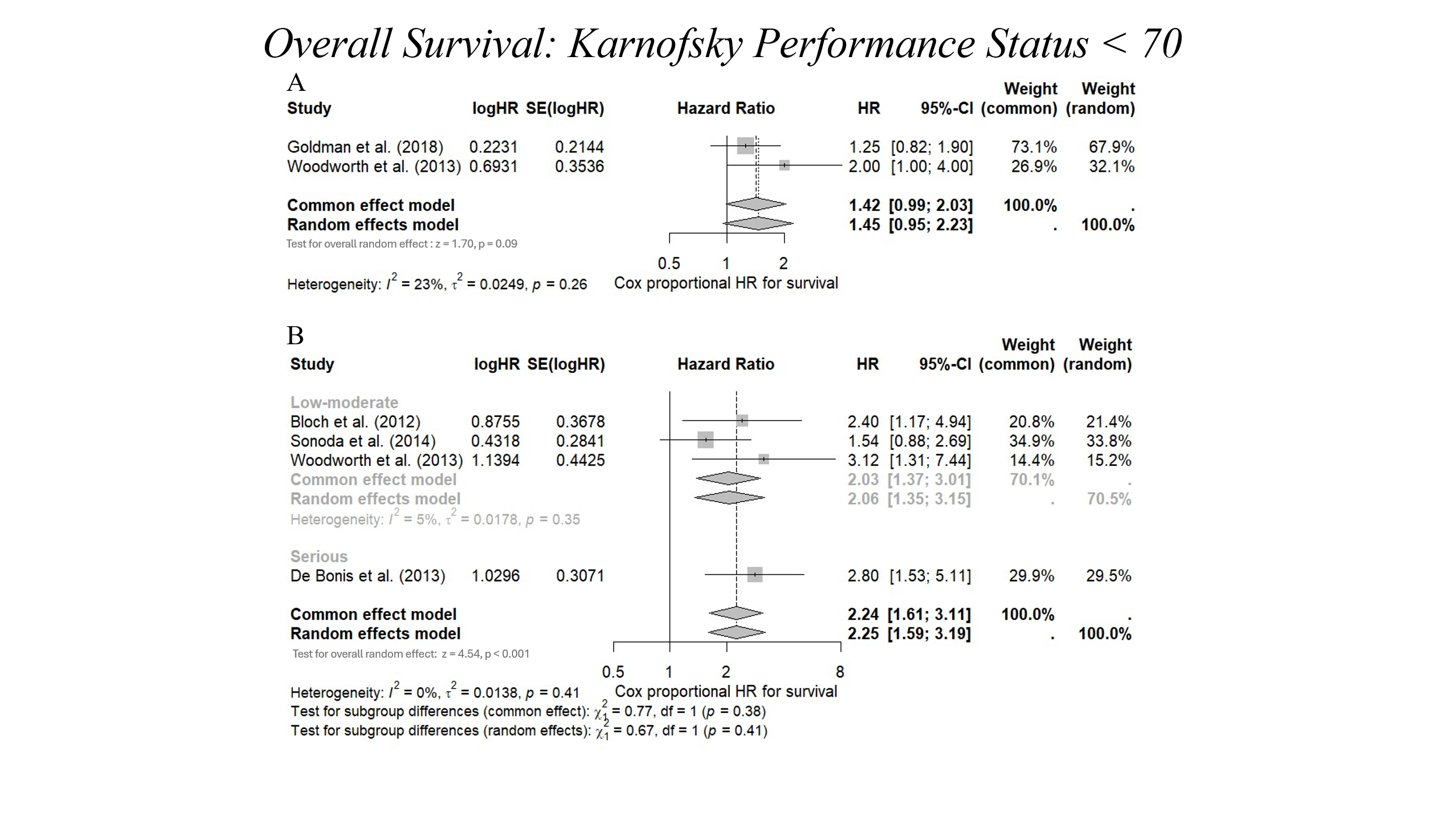


**(A) Supplementary Figure 3A is a forest plot indicating the univariate cox proportional hazard ratio representing the association between a Karnofskyperformance status (KPS) score of less than 70 and survival after re-resection. (B) Supplementary Figure 3B is a forest plot indicating the multivariate cox proportional hazard ratio representing the association between a Karnofsky performance status (KPS) score of less than 70 and survival after re-resection, with studies divided into subgroups based on the overall risk of bias scored based on the ROBINS-I tool.** A hazard ratio < 1.00 indicates association with increased survival, whereas a hazard ratio >1.00 indicates association with worse survival. The weighting of each study is derived from the inverse of the variance of each study’s estimate hazard ratio. The size of the grey square is inversely proportional to the standard error, and the straight line indicates the 95% confidence intervals, which are shown in the square brackets. The diamonds indicate the overall pooled hazard ratio, and the random effects model is reported as the outcome. Heterogeneity is indicated by the I^2^ and tau^2^ values. P value <0.05 is deemed significant. Furthermore, for every study the following are displayed: study author with publication date (“Study”), HR, log(HR), the standard error of logHR (SElog(HR)), 95% confidence intervals, and the weighting of each study in percentage (%). A significant pooled hazard ratio (2.25) was only found with forest plot synthesis of multivariate hazard ratios, indicating association with worse survival with a pre-operative KPS of less than 70. Heterogeneity was not statistically significant (p=0.26 for univariate, p=0.41 for multivariate).

# **Supplementary Figure 4:** Forest plots showing univariate and multivariate cox HR for KPS < 80.


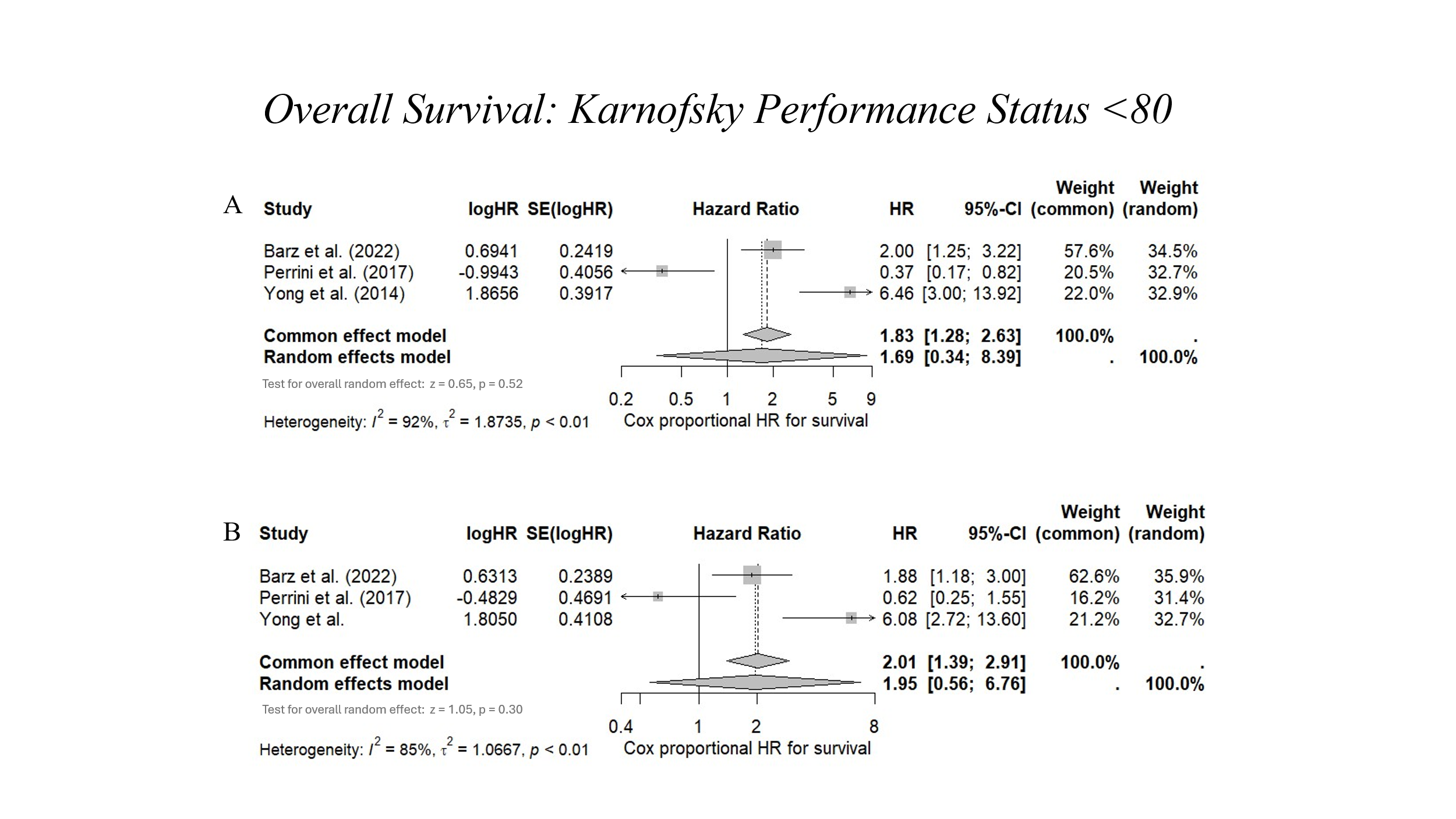


**(A) Supplementary Figure 4A is a forest plot indicating the univariate cox proportional hazard ratio representing the association between a Karnofsky performance status (KPS) score of less than 80 and survival after re-resection. (B) Supplementary Figure 4B is a forest plot indicating the multivariate cox proportional hazard ratio representing the association between a Karnofsky performance status (KPS) score of less than 80 and survival after re-resection.** A hazard ratio < 1.00 indicates association with increased survival, whereas a hazard ratio >1.00 indicates association with worse survival. The weighting of each study is derived from the inverse of the variance of each study’s estimate hazard ratio. The size of the grey square is inversely proportional to the standard error, and the straight line indicates the 95% confidence intervals, which are shown in the square brackets. The diamonds indicate the overall pooled hazard ratio, and the random effects model is reported as the outcome. Heterogeneity is indicated by the I^2^ and tau^2^ values. P value <0.05 is deemed significant. Furthermore, for every study the following are displayed: study author with publication date (“Study”), HR, log(HR), the standard error of logHR (SElog(HR)), 95% confidence intervals, and the weighting of each study in percentage (%). An insignificant pooled hazard ratio for older age was found in both univariate and multivariate forest plot analyses. Heterogeneity was statistically significant (p<0.01).

# **Supplementary Figure 5:** Forest plots showing univariate and multivariate cox HR for Age including studies with recurrence in only IDH-wildtype glioblastoma.


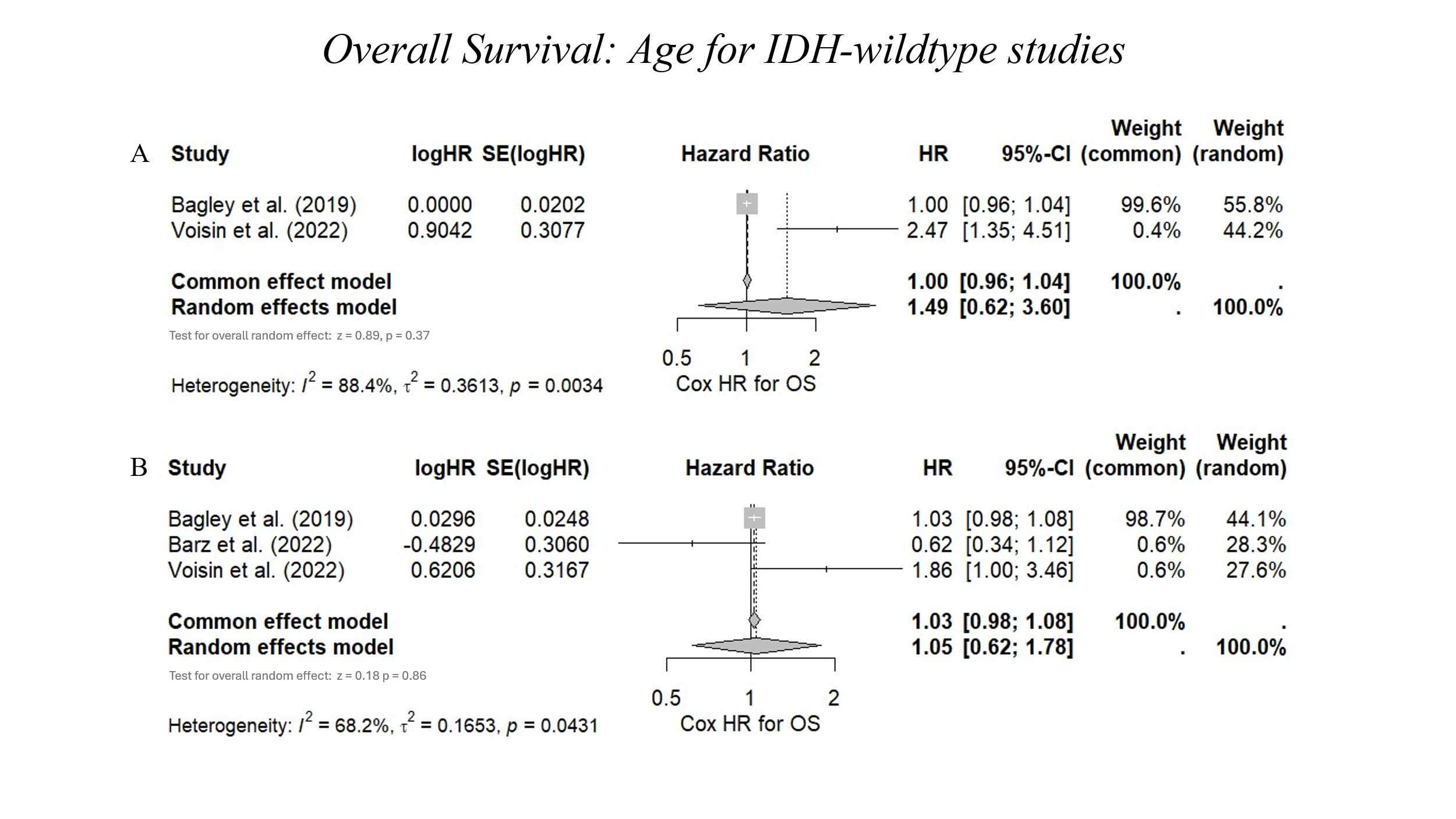


**(A) Supplementary Figure 5A is a forest plot indicating the univariate cox proportional hazard ratio representing the association between age and survival after re-resection for studies including only patients undergoing re-resection for recurrent IDH-wildtype glioblastoma. (B) Supplementary Figure 5B is a forest plot indicating the multivariate cox proportional hazard ratio representing the association between age and survival after re-resection for studies including only patients undergoing re-resection for recurrent IDH-wildtype glioblastoma.** A hazard ratio < 1.00 indicates association with increased survival, whereas a hazard ratio >1.00 indicates association with worse survival. The weighting of each study is derived from the inverse of the variance of each study’s estimate hazard ratio. The size of the grey square is inversely proportional to the standard error, and the straight line indicates the 95% confidence intervals, which are shown in the square brackets. The diamonds indicate the overall pooled hazard ratio, and the random effects model is reported as the outcome. Heterogeneity is indicated by the I^2^ and tau^2^ values. P value <0.05 is deemed significant. Furthermore, for every study the following are displayed: study author with publication date (“Study”), HR, log(HR), the standard error of logHR (SElog(HR)), 95% confidence intervals, and the weighting of each study in percentage (%). An insignificant pooled hazard ratio for older age was found in both univariate and multivariate forest plot analyses. Heterogeneity was statistically significant (p=0.003; p=0.043).

# **Supplementary Figure 6:** Forest plots showing univariate and multivariate cox HR for time to recurrence/re-resection (TTR) including studies with recurrence in only IDH-wildtype glioblastoma.


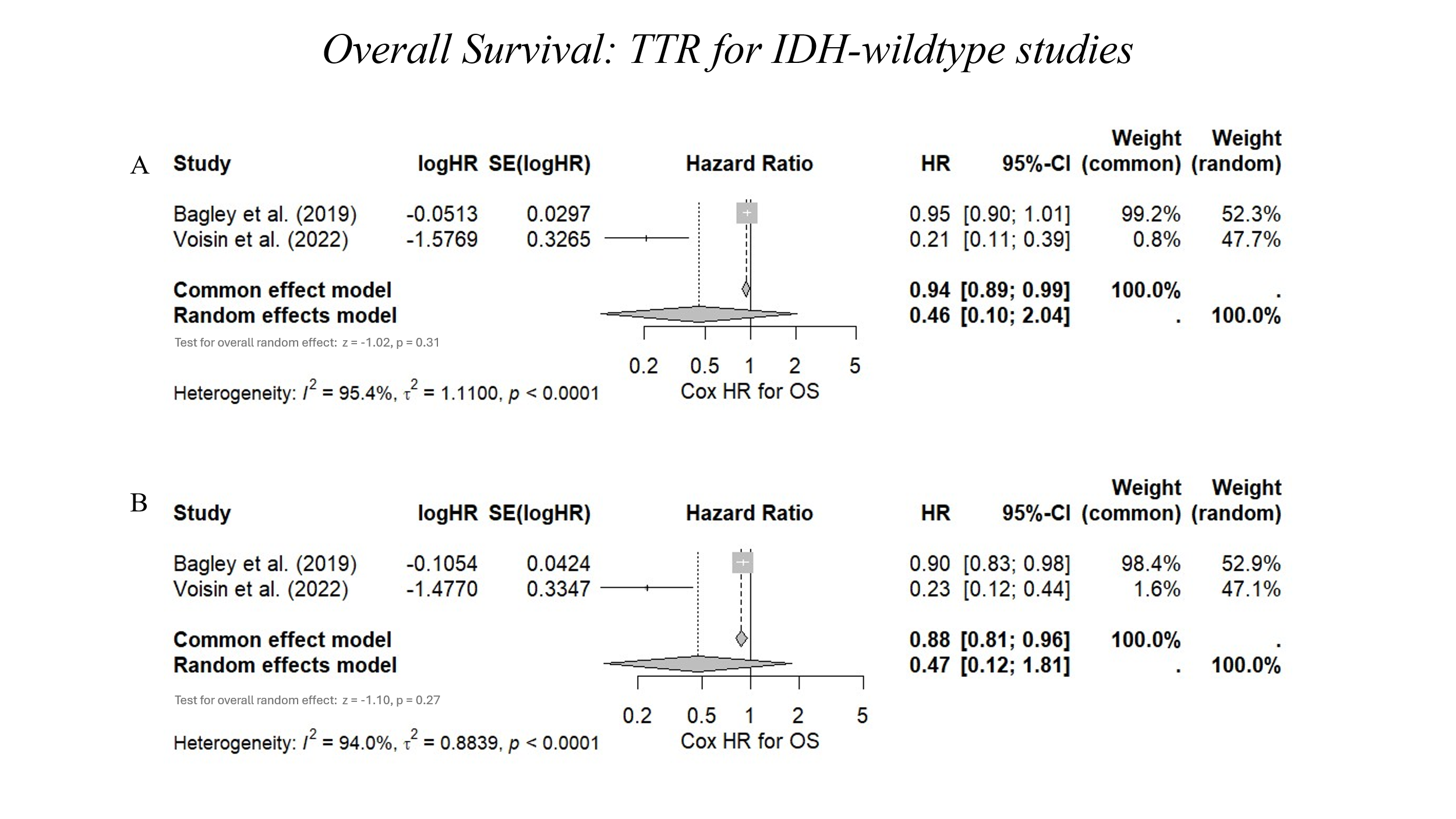


**(A) Supplementary Figure 6A is a forest plot indicating the univariate cox proportional hazard ratio representing the association between TTR and survival after re-resection for studies including only patients undergoing re-resection for recurrent IDH-wildtype glioblastoma. (B) Supplementary Figure 6B is a forest plot indicating the multivariate cox proportional hazard ratio representing the association between TTR and survival after re-resection for studies including only patients undergoing re-resection for recurrent IDH-wildtype glioblastoma.** A hazard ratio < 1.00 indicates association with increased survival, whereas a hazard ratio >1.00 indicates association with worse survival. The weighting of each study is derived from the inverse of the variance of each study’s estimate hazard ratio. The size of the grey square is inversely proportional to the standard error, and the straight line indicates the 95% confidence intervals, which are shown in the square brackets. The diamonds indicate the overall pooled hazard ratio, and the random effects model is reported as the outcome. Heterogeneity is indicated by the I^2^ and tau^2^ values. P value <0.05 is deemed significant. Furthermore, for every study the following are displayed: study author with publication date (“Study”), HR, log(HR), the standard error of logHR (SElog(HR)), 95% confidence intervals, and the weighting of each study in percentage (%). An insignificant pooled hazard ratio for older age was found in both univariate and multivariate forest plot analyses. Heterogeneity was statistically significant (p<0.001).

# References

(1) Page MJ. et al. The PRISMA 2020 statement: an updated guideline for reporting systematic reviews. *BMJ.* 2021; **372** n71. 10.1136/bmj.n71.

(2) Glasziou P. et al. Oxford Centre for Evidence-Based Medicine 2011 Levels of Evidence.

(3) Sterne JA. et al. *ROBINS-I: a tool for assessing risk of bias in non-randomised studies of interventions.* BMJ; 2016.

(4) De Bonis P. et al. The impact of repeated surgery and adjuvant therapy on survival for patients with recurrent glioblastoma. *Clinical neurology and neurosurgery.* 2013; **115** (7): 883-6. 10.1016/j.clineuro.2012.08.030.

(5) Mandl ES, Dirven CMF, Buis DR, Postma TJ, Vandertop WP. Repeated surgery for glioblastoma multiforme: only in combination with other salvage therapy. *Surgical neurology.* 2008; **69** (5): 506-509. 10.1016/j.surneu.2007.03.043.

(6) Park C. et al. A practical scoring system to determine whether to proceed with surgical resection in recurrent glioblastoma. *Neuro-oncology.* 2013; **15** (8): 1096-101. 10.1093/neuonc/not069.

(7) Woodroffe RW. et al. Survival after reoperation for recurrent glioblastoma. *Journal of Clinical Neuroscience.* 2020; **73** 118-124. 10.1016/j.jocn.2020.01.009.

(8) Yong RL. et al. Residual tumor volume and patient survival following reoperation for recurrent glioblastoma. J Neurosurg. 2014; **121** (4): 802-9. doi: 10.3171/2014.6.JNS132038.

(9) McGuinness LA, Higgins JPT. Risk-of-bias VISualization (robvis): An R package and Shiny web app for visualizing risk-of-bias assessments. *Research Synthesis Methods.* 2020; n/a 10.1002/jrsm.1411.
